# Quantum-accurate machine learning potentials for metal-organic frameworks using temperature driven active learning

**DOI:** 10.1038/s41524-024-01427-y

**Published:** 2024-10-08

**Authors:** Abhishek Sharma, Stefano Sanvito

**Affiliations:** School of Physics, AMBER and CRANN Institute, Trinity College, Dublin 2, Ireland

**Keywords:** Metal-organic frameworks, Electronic structure

## Abstract

Understanding structural flexibility of metal-organic frameworks (MOFs) via molecular dynamics simulations is crucial to design better MOFs. Density functional theory (DFT) and quantum-chemistry methods provide highly accurate molecular dynamics, but the computational overheads limit their use in long time-dependent simulations. In contrast, classical force fields struggle with the description of coordination bonds. Here we develop a DFT-accurate machine-learning spectral neighbor analysis potentials for two representative MOFs. Their structural and vibrational properties are then studied and tightly compared with available experimental data. Most importantly, we demonstrate an active-learning algorithm, based on mapping the relevant internal coordinates, which drastically reduces the number of training data to be computed at the DFT level. Thus, the workflow presented here appears as an efficient strategy for the study of flexible MOFs with DFT accuracy, but at a fraction of the DFT computational cost.

## Introduction

Compounds presenting nanometer-size voids form a promising materials platform for various applications, including selective gas diffusion, adsorption and catalysis^1,2^. Flexible metal-organic frameworks (MOFs) have emerged as an intriguing class of nanoporous materials, which allow one to dynamically tune and control the structure and properties of such voids^3–5^. MOFs are crystalline materials made through reticular chemistry, where organic linkers are connected to metal units via coordination bonds. The flexibility of MOFs, in combination with external stimuli, affects the pores and pore channels and gives rise to interesting properties such as linker rotation, gate opening, swelling, negative thermal expansion, negative adsorption etc^3,6–8^.

In order to study computationally the effects of an external stimulus, such as pressure and temperature, on the properties of flexible MOFs, a detailed analysis of the framework dynamics at an extended length and time scale is necessary^4,9–12^. This can be performed through molecular dynamics (MD) simulations. Ab-initio MD (AIMD), as implemented for instance with density functional theory (DFT), provides the most accurate estimation of the potential energy surface (PES). However, the computational overheads are significant and hence AIMD simulations are usually limited to few hundreds of atoms and pico-second time scales. Alternatively, one can use classical interatomic potential models or force-fields. These approximate the PES of a material with the help of parametric functions and may provide estimates of energy, forces, and virial-stress of thousand-atom atomic configurations in a short time. However, the use of classical force-fields for MOFs is hampered by their poor performance with atomic environments presenting coordination bonds^13^. Despite this limitation, a variety of classical force-fields have been used to study the properties of MOFs^14–23^. These force-fields are either transferrable (e.g., UFF^14^, DREIDING^17^, UFF4MOF^15,16^ etc.) or developed for a specific MOF (e.g., QUICK-FF^19^ and MOF-FF^21,23^).

A possible strategy to achieve DFT accuracy at a computational cost comparable to that of classical force fields is provided by machine-learning potentials (MLPs)^24–30^. In these, the atomic chemical environments are represented by mathematical descriptors at various levels of complexity^31^, while the corresponding fitting parameters are obtained by training appropriate machine-learning models over the energy, forces, and virial-stress values of a large number of configurations. These are typically obtained by DFT. The accuracy of a MLP to predict the PES of a material depends on the chemical-environment descriptors, the number of parameters in the model, the size and diversity of the training set, and the training procedure.

Recently several computational works have used MLPs to study MOFs^12,32–42^. Most of these employ neural-network potentials (NNPs), which require thousands of training configurations and comprise hundred thousands of parameters to fit. In most of the earlier works, the training configurations were generated via picosecond-long AIMD simulations, which require a long computational time and significant computational resources. Furthermore, the fit of the many parameters is also computationally intensive and the model are little interpretable, namely it is not simple to define from the outset the boundary of the model’s validity (e.g., the temperature and pressure range).

**Fig. 1:**
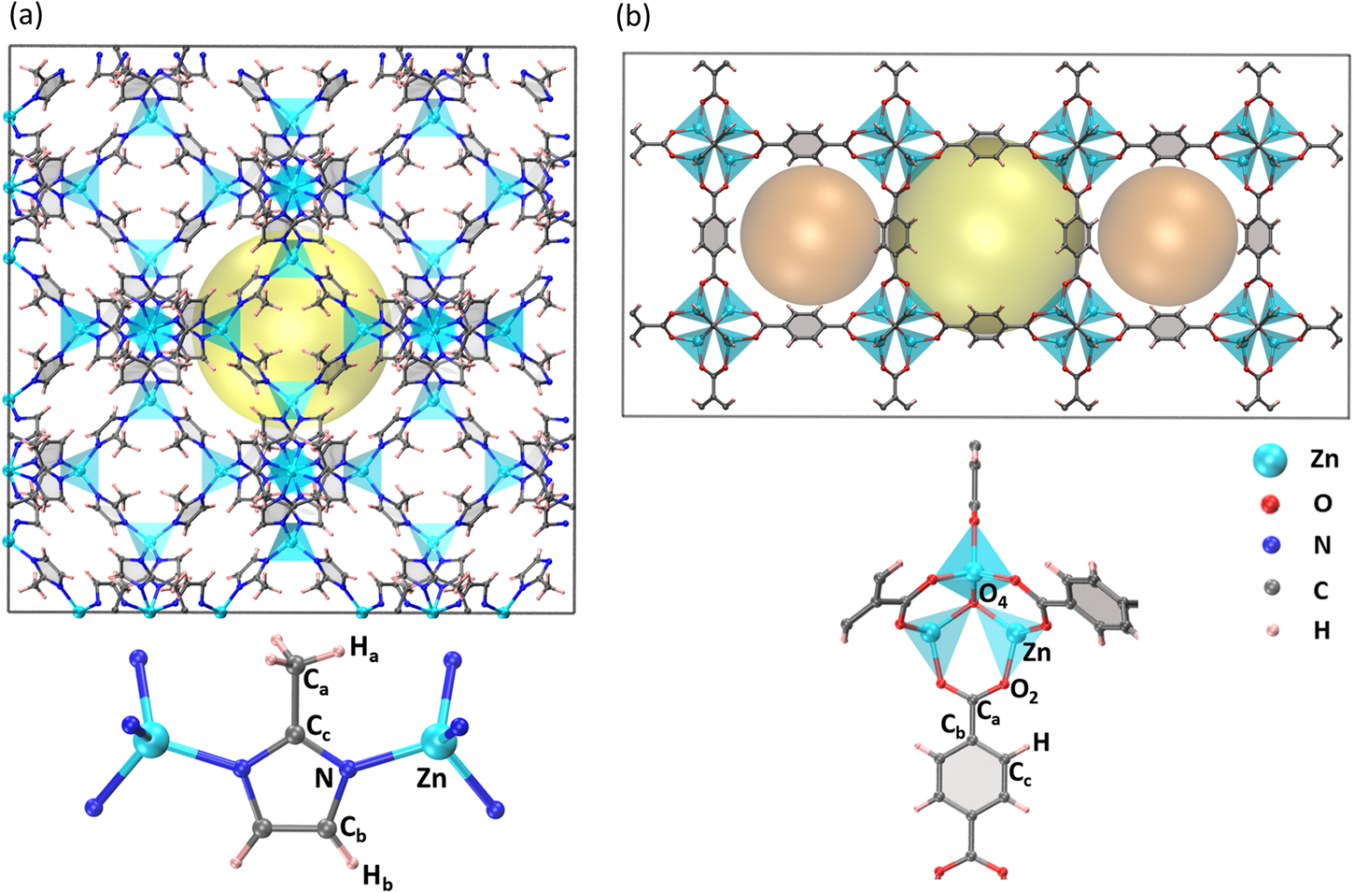
Atomic structures of the MOFs investigated in this work. **a** ZIF-8 and (**b**) MOF-5 (cyan: zinc, blue: nitrogen, red: oxygen, gray: carbon, pink: hydrogen). The basic building units with atom types (C_a_, C_b_, etc.) are shown below the main structures. In order to show clearly the pores (yellow and orange spheres), supercells of size 2 × 2 × 2 and 1 × 1 × 2 are shown for ZIF-8 and MOF-5, respectively. In all calculations described in this work, the unit cell of both ZIF-8 (containing 276 atoms) and MOF-5 (containing 424 atoms) are used.

In this work, we use the spectral neighbor analysis potential (SNAP)^43^ as MLP model to study the structural and vibrational properties of MOFs at finite temperature and pressure. SNAP was previously shown to perform well for organic molecules and coordination complexes, and thus it appears as a natural choice for MOFs^44^. SNAP is based on many-body descriptors and linear models, hence, when compared to neural-network potentials, it requires only a few hundreds of parameters to obtain similarly accurate fits. For this reason, SNAP typically demands a much smaller training set than those needed by neural-networks, so that a limited number of DFT calculations is necessary. As a test bench, here we develop two SNAPs for the widely studied ZIF-8^45^ and MOF-5^46^ MOFs (their structures are shown in Fig. 1). These two particular MOFs have been selected for our study, since various experimental results are available, so that the validity of our approach can be thoroughly tested.

Firstly, we perform a very detailed analysis of the SNAP learning curves, which allows us to propose a protocol for generating correlation-free training sets. Then, we compute various structural and vibrational properties as a function of temperature, and we compare them with available experimental data, demonstrating an excellent agreement. Although applied here to ZIF-8 and MOF-5, our approach is completely general and widely applicable to any other MOFs, whose electronic structure is accessible by DFT (or other ab initio electronic structure methods). This allows one to develop predictive SNAPs for MOFs by using only a few hundreds DFT calculations and a simplified training procedure.

## Results

### Active learning algorithm

The construction of an adequate training set is crucial for the formulation of a MLP. In fact, MLPs are not physically informed, so that their knowledge of the energy and forces of a particular molecular structure is rooted in having been trained on structures that contain similar local environments. Importantly, in general, MLPs are not guaranteed to extrapolate to poorly known configurations, for which they can catastrophically fail. As such, an ideal training set needs to contain all the local environments that the system will experience when performing the inference, for instance the ones explored during MD simulations. Such training set should also be finely balanced, namely, even when complete, it should not be dominated by a particular pool of local environments. Finally, the size of the training set must be kept as limited as possible, so that the construction of the MLP itself will be numerically convenient in the computational economy of the workflow that one wants to pursue.

With all these requirements in mind we present here an active-learning strategy that allows us to construct a balanced training set, while performing a limited number of DFT calculations. This consists of two main tasks, namely (1) an algorithm that maps the diversity of the training set and ensures that all the relevant local environments are represented, and (2) a strategy to generate the molecular configurations containing those environments. The algorithm is then used for both ZIF-8 and MOF-5, although in two different ways. In fact, although in both cases the first step is identical, then for ZIF-8 the configurations are generated with AIMD, while for MOF-5 they originate from MD runs performed at increasingly large temperatures with subsequently more refined SNAPs. This is because we effectively use the construction of the ZIF-8 SNAP as a learning step to the construction of an efficient method, which is then used for MOF-5.

### Selection of the configurations to include in the training set

**Fig. 2:**
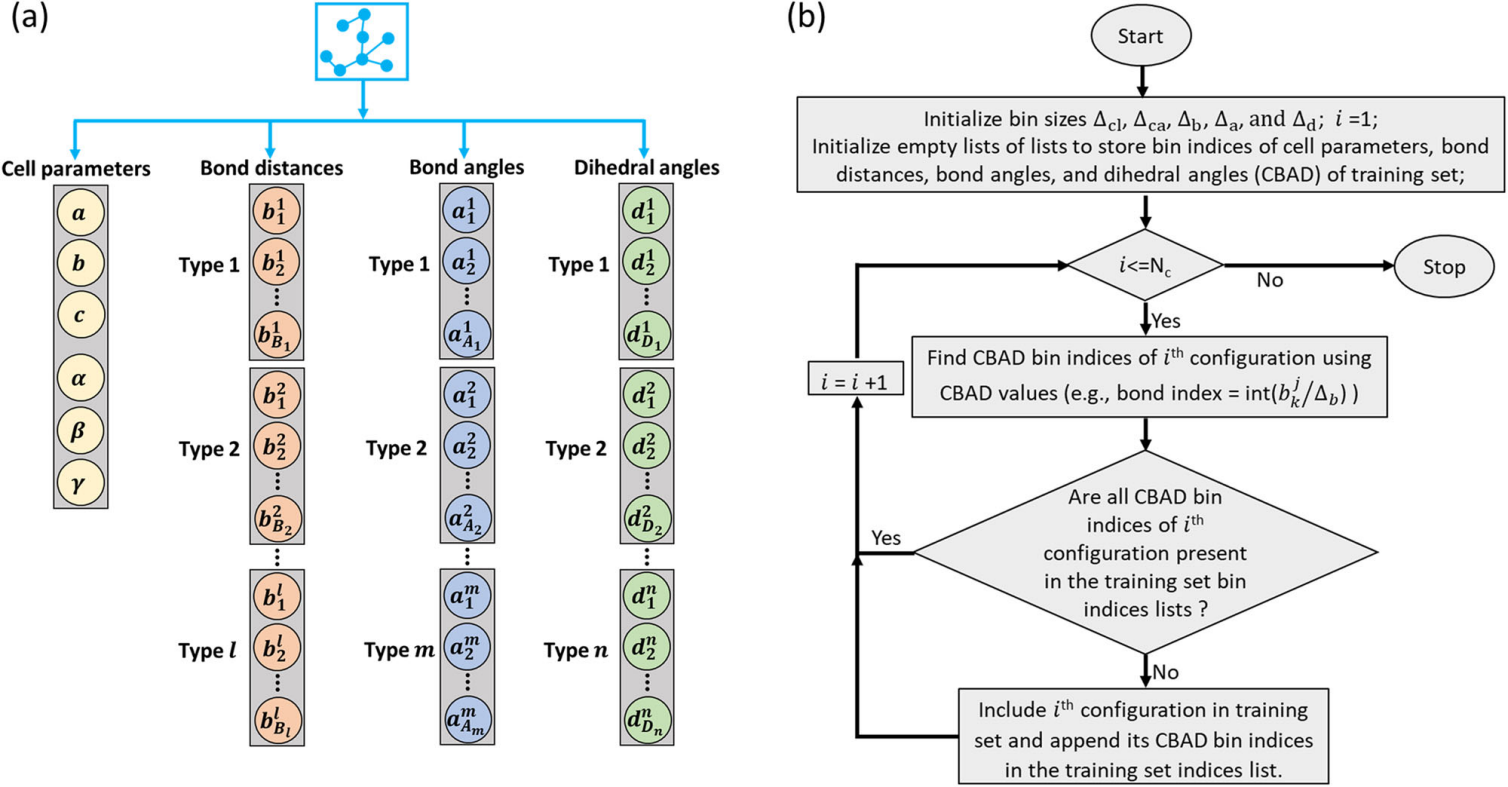
Outline of the active learning algorithm developed in this work. **a** Schematic showing the cell parameters (lattice parameters: $a$, $b$, $c$; cell angles: $\alpha$, $\beta$, $\gamma$) and different types of bonds, angles, and dihedrals present in the atomic configuration of a MOF structure. This representation of an atomic configuration is termed here as CBAD (cell parameters, bonds, angles, and dihedrals). **b** A flowchart illustrating our CBAD-based algorithm to select training set configurations from a given set of *N*_*c*_ configurations. Here ${\Delta }_{{\rm{cl}}}$, ${\Delta}_{{\rm{ca}}}$, ${\Delta }_{{\rm{b}}}$, ${\Delta }_{{\rm{a}}}$, and ${\Delta }_{{\rm{d}}}$ are the descriptor resolutions for the cell lengths ($a$, $b$, $c$), cell angles ($\alpha$, $\beta$, $\gamma$), bond distances (${b}_{k}^{j}$), angles (${a}_{k}^{j}$), and dihedral angles (${d}_{k}^{j}$), respectively. The ${\Delta }_{{\rm{i}}}$ values are used to find CBAD bin indices (e.g., bond index = int(${b}_{k}^{j}/{\Delta }_{b}$)). The algorithm then compares the CBAD indices of a new configuration with the CBAD indices of the training set and will include the new configuration in training set, if at least one of the indices is not present in the configuration matrix.

The atomic configuration of a MOF structure can be defined through the knowledge of the unit cell parameters, the atomic bonds, the bond angles, and the dihedral angles [see Fig. 2a]. Classical force-fields use this information to compute the energy and forces of a configuration in terms of non-bonded and bonded interactions. In the case of non-bonded interactions, atoms are classified into different types, based on their chemical identity and connectivity, information which is then used to define the interaction parameters (e.g., electrostatic charges, Lennard-Jones parameters, etc.). Similarly, bond lengths, bond angles and dihedral angles are classified into different types and correspondingly interaction parameters of the bonded interaction are thus computed.

Inspired by this structure-informed approach, we have developed a simple algorithm to track the diversity and relevance of the local atomic environments included in a training set [see Fig. 2b for details]. As for the classical force fields, we would like to differentiate structures in terms of a limited number of structural descriptors, namely the cell-parameters, bonds, angles, and dihedrals (collectively called CBAD). The total number of structure descriptors, ${N}_{{CBAD}}$, depends on the MOF of interest and consists of 6 cell parameters, $l$ bond types, $m$ angle types, and $n$ dihedral angle types [see Fig. 2a for details]. Then, we define the resolution of each descriptor, $\Delta$, which is the minimum difference between two values of the descriptor that one can distinguish. This allows us to represent each value of the descriptors as an integer (representing bin index), namely as int($\theta /\Delta$), where $\theta$ is the value of the descriptor. Each MOF configuration has multiple values of each descriptor (except the 6 cell parameters, which have only one value). Therefore, for each MOF configuration we track ${N}_{{CBAD}}$ lists of integers, where each list can have multiple integer values. If we wish to consider *M* possible values for each descriptor in the training set, we will have ${M}^{{N}_{{CBAD}}}$ possible configurations (or configuration matrix) to explore in a ${N}_{{CBAD}}$ dimensional configuration space. The question is now how to populate such space with relevant configurations. In principle, one can generate MOF configurations where the descriptors are varied one at a time, but this will necessitate ${M}^{{N}_{{CBAD}}}$ DFT calculations, a number that can be prohibitively large. The strategy chosen here, instead, is that of mapping the entire space with a limited number of configurations.

In order to reduce the number of DFT calculations, we consider the multiplicity of descriptor values in an atomic configuration of MOF (within the ${N}_{{CBAD}}$ lists of integers). If an atomic configuration results from a MD simulation (at certain temperature), then all values of a descriptor in that configuration would be different and belong to a distribution (of descriptor at certain temperature). In such configuration, the atoms in that configuration can have multiple local chemical environments. Thus, instead of tracking the ${M}^{{N}_{{CBAD}}}$ configuration matrix, we individually track ${N}_{{CBAD}}$ descriptors and corresponding configuration matrix of size of ${N}_{{CBAD}}\times M$. With this simplification, our algorithm proceeds as follows. An initial configuration defines multiple value for each of the *N*_CBAD_ descriptors, we consider only unique values for each descriptor and populate the values in the *N*_CBAD_ descriptor lists (to sample the *N*_CBAD_$\times$*M* configuration matrix). The next configuration will then define a new *N*_CBAD_ list of descriptors. If at least one of them is not already present in the corresponding descriptor lists, then such configuration will be accepted and it will be part of the training set. In this way we ensure that all the relevant values of each descriptor, within a precision $\Delta$, are represented at least once in our training set. A schematic illustration of the algorithm is provided in Fig. 2b, while a detailed pseudocode is given in the Supplementary Fig. 1. It is to be noted that this CBAD algorithm selects configurations based on the order they are presented. Thus, if CBAD is applied individually to two different set of configurations which are in different order with same atomic structures (e.g., set1 = [C_1_, C_2_, C_3_, C_4_, C_5_] and set2 = [C_4_, C_2_, C_5_, C_1_, C_3_]), than it will select different type of training set configurations (e.g., [C_1_, C_2_, C_4_] from set1 and [C_4_, C_5_, C_3_] from set2). Furthermore, the CBAD algorithm can identify the diversity between a typical MD simulation in the NVT/NPT ensemble and a biased simulation (e.g., metadynamics), since the distributions of the CBAD values are different in these two cases.

### Training the SNAP

Within the SNAP formalism^43^ the total energy, $E$, of a molecule (or a solid) is expressed as the sum of individual atomic energies, ${E}_{i}$. These are, in turn, function of the local chemical environment of each individual atom, which is defined within a radial cut-off. SNAP then expands the local atomic-density distribution over four-dimensional spherical harmonics and constructs the associated bispectrum components, ${B}_{j}$, which form a rotationally invariant set of descriptors. Finally, the atomic energies are taken as a linear function of the bispectrum components, namely

$$E=\mathop{\sum }\limits_{i}^{{N}_{a}}{E}_{i}=\mathop{\sum }\limits_{i}^{{N}_{a}}\mathop{\sum }\limits_{l}^{{N}_{2J}}{\beta }_{i}^{l}{B}_{i}^{l}$$

where, ${N}_{a}$ is total number of atoms, ${N}_{2J}$ is total number of bispectrum functions ($2J$ controls the order of the expansion), and ${\beta }_{i}^{l}$ are the coefficients of the bispectrum components. The accuracy of the chemical-environment description can be tuned by tuning the number of bispectrum components. Earlier work^44^ has shown that considering 56 bispectrum components (corresponding to $2J$ = 8) per chemical species results in a reasonable accuracy, and thus we have used same value here. In both ZIF-8 and MOF-5 there are 7 different atom types (see Fig. 1 – note that C, O, and H atoms with different coordination are considered as different atom types), so that our SNAP models are constructed over 392 bispectrum functions (and 392 ${\beta }_{i}^{l}$ values). The SNAP training, namely the computation of the ${\beta }_{i}^{l}$ values, is here performed over the energy, forces and the virial-stress of each of the configurations contained in the training set, with the reference values being computed with DFT (see Methods section for details). Thus, each configuration provides 3${N}_{a}$ + 7 training data (1 energy, 3${N}_{a}$ forces, and 6 virial-stress components). The unit cells of ZIF-8 and MOF-5 contain 276 and 424 atoms, respectively. Therefore, if the training set comprises in the region of 600 configurations, we will have approximately 0.5 and 0.7 million of training data for ZIF-8 and MOF-5, respectively. We generate the bispectrum components of a given configuration by using the Large-scale Atomic/Molecular Massively Parallel Simulator (LAMMPS)^47,48^ package and then obtain the bispectrum coefficients through ridge regression. We optimize the SNAP hyperparameters (atomic-species-dependent cutoff radius and chemical-species weights) by using the Scipy package and we drive the optimization by minimizing the error over energy, forces and stress tensor. Then, the SNAP training is performed at the optimal hyperparameters and the final model is used to perform MD simulations.

### Generation of the training and test sets for ZIF-8

In order to establish a general SNAP-training protocol for MOFs, we have first developed the potential for ZIF-8. The unit cell of ZIF-8 contains four elements (C, H, N, and Zn), 7 atom types [see Fig. 1a] and 276 atoms. The different configurations to be included in the training set are generated by first following the computationally intensive approach used in earlier works, namely we perform AIMD simulations (details are given in Methods section). These have a duration of 1 ps (with a 0.5 fs timestep) at temperatures ranging from 100 K to 1000 K with a 100 K interval. From the generated 20,000 configurations, we then select those to include in the training set by using the simple algorithm described in the previous section. Note that for all configurations included in the training and test set, we run high-quality DFT calculations with the higher cut-off of 1000 Ry (details of all DFT calculations are given in Methods section).

In general, the number of configurations contained in the training set should be optimal, since a few configurations will result in poor a representation of the PES, while too many configurations are associated to a high computational cost and to a possible imbalance in the representation of the main structural characteristics of the MOF. Thus, finding the optimal number of configurations is an essential step for the development of the MLP. Our strategy to populate the configuration matrix mitigates the risk of oversampling, and we can systematically change the number of configurations by changing the resolution of the structural descriptors (how finely we sample each descriptor). In this way we create training sets ranging from 50 to 3000 configurations and fit a SNAP for each of these training sets. Then, the performance test is conducted over two different sets. The first, referred here as test set A, contains around 5000 configurations obtained from AIMD simulations (at different temperatures between 100 to 500 K), while the second (test set B) is generated by using classical MD simulations (using force-field proposed by Weng et al.^22^) of the ZIF-8 unit cell at 500 K. In this second case we select around 2000 configurations.

**Fig. 3:**
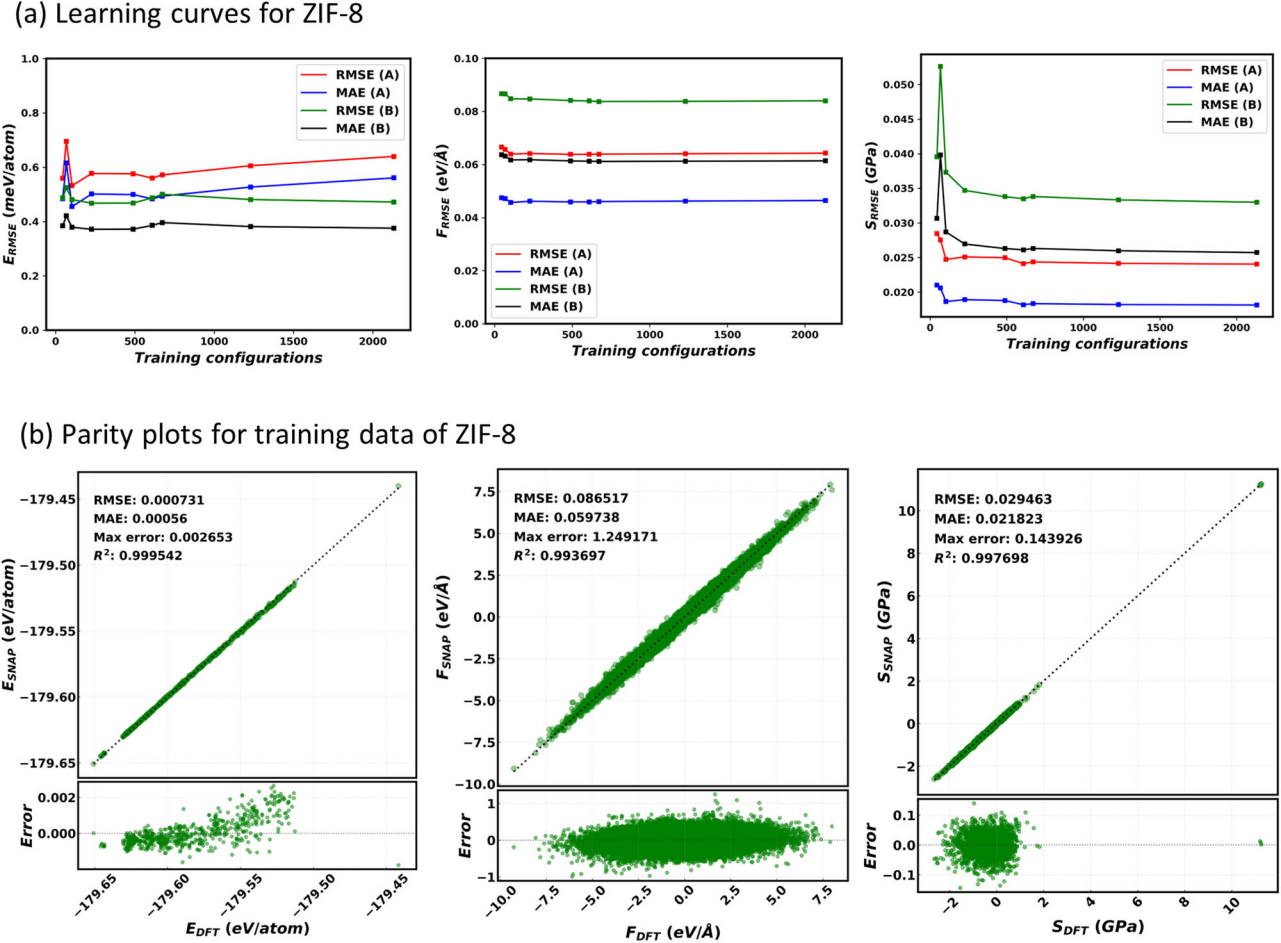
Performance of the trained SNAP model for ZIF-8. **a** Learning curves for the RMSE and MAE for energy (left-hand side panel), forces (middle panel) and virial-stress (right-hand side panel). Data are presented for test set A (composed of ~5000 configurations from AIMD simulations) and test set B (composed of ~2000 configurations from classical MD simulations) as a function of the number of configurations in the training set. Note that no significant change in the error is observed after the training set size reaches ~ 600 configurations. **b** Parity plots for energy, forces and virial-stress values comparing DFT and SNAP (trained over 672 configurations) values. The RMSE is 0.7 meV/atom, 86 meV/Å, and 29.5 MPa, respectively for energy, forces and virial-stress.

Depending on the training set size and diversity, a part of the PES (or the configuration space) can be represented more accurately (less error), less accurately (moderate but acceptable error), or unphysically (very high error). In order to study the effect of the training set size and diversity, we compute the SNAP learning curves for ZIF-8 (shown in Fig. 3). To obtain the learning curves, we create training sets of different sizes using different values of the descriptor resolutions (details are given in Supplementary Table 1). The learning curves are taken over the diverse test set and display both the root mean square error (RMSE) and the mean absolute error (MAE) as a function of the number of configurations in the training set. With a very small number of configurations, we observe high errors in the energy, forces and the virial-stress learning curves, signaling an unphysical representation of the PES. As the size of the training set increases, we observe a decrease in errors, indicating improvement in the representation of the PES. For training sets containing in excess of 600 configurations an error plateau is found, indicating that the PES is well described. This implies that 600 configurations are optimal for a good representation of the PES for a MOF like ZIF-8. Including more diverse configurations can further improve the representation of unexplored regions of the PES. Thus, after the plateau in the learning curves, the errors on the test set can decrease if the training set size is increased by adding configurations from a region close to the PES where test configurations are distributed. Interestingly, the errors can also increase if the additional configurations included are either far from the test set configurations (representing other portion of the PES) or have some level of correlation. Presence of correlation lowers the diversity in the training set and populates unevenly a particular region of the PES, a fact that may result in overfitting that region of the PES.

Here, for the test set A we observe a marginal error enhancement in energy (around 1 meV) when the configurations are increased beyond 600, a feature that may suggest minor overfitting. It is to be noted that, configurations are selected sequentially from a pool of 1 ps AIMD simulation trajectories, a selection that may be affected by correlation among the configurations (details are given in Supplementary Fig. 4) and this could be the possible reason of overfitting. For further MD simulations of ZIF-8 we use the current training set containing the selected 672 configurations; the associated parity plots, computed over energy, forces and viral stress, are displayed in Fig. 3. However, in order to check the cause of the overfitting, we have performed a new analysis, where we shuffle the order of the AIMD configurations (to disrupt correlation) and reselect the training set, using the CBAD algorithm. The learning curves for this new training process are shown in Supplementary Fig. 5, where we found that the marginal error enhancement in the energy of the test set A vanishes and a plateau in all energy, forces, and stress error values is observed beyond the 600 training configurations. In any case, the errors of the converged SNAP are extremely low, namely of the order of 0.5 meV/atom, 50 meV/Å and 25 MPa, respectively for energy, forces and stress tensor. This level of accuracy is certainly enough to perform reliable MD over a broad temperature range, as we will demonstrate later on.

### Training and Test Set for MOF-5

**Fig. 4:**
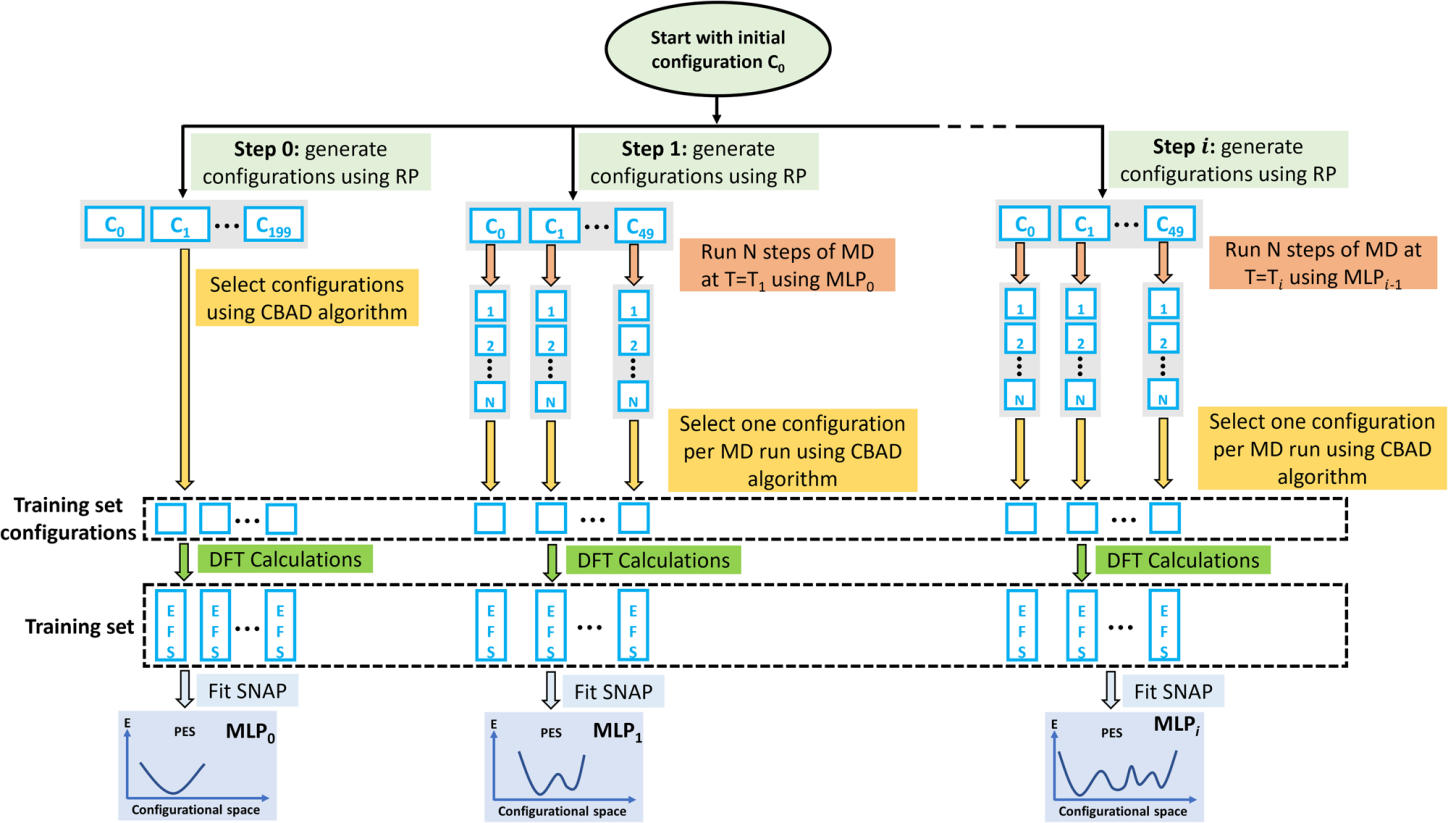
Overview of the computational workflow used to generate the training set for MOF-5. At each step a new SNAP is constructed by using the structure configurations obtained from the MD simulations performed with the SNAP trained at the previous step (see details in the text). ‘RP’ means random perturbation of the atomic positions.

In the construction of the ZIF-8 SNAP we did generate about 20,000 configurations, but then realised that 600 are ideal to fit a high-performing model. Now, for MOF-5 we wish to establish a method that allows us to compute only the 600 configurations needed without any redundancy. In a recent work, an incremental learning approach was used in combination with metadynamics to generate the training set configurations of a MLP^36^. In a metadynamics simulation, a few collective variables are defined and bias is added along their trajectories to explore a particular region of the phase space. Since increasing the temperature corresponds to enlarging the phase space explored for all structural descriptors (and not just the collective variables), here we develop a simple algorithm driven by temperature to generate the training set for MOFs (see Fig. 4 for details).

The proposed algorithm proceeds as following (see Fig. 4). Firstly, we take the experimental crystal structure and generate different configurations by introducing a small random perturbation to the atomic positions (Step 0 in Fig. 4). Among these configurations we select those to populate the defined configuration matrix according to the CBAD algorithm described before, and their electronic structure is computed by DFT. The DFT energies, forces and stress tensors are then used to train an initial SNAP (MLP_0_). The following step (Step 1 in Fig. 4) performs 50 independent MD runs, starting from 50 inequivalent configurations obtained by random displacing atoms from the experimental structure. The MD is conducted, starting from different initial velocities, at the low temperature of 100 K (in the *NPT* ensemble) by using MLP_0_ for approximately 2000 steps. We then select at most one configuration from each MD run to be included in the training set, according to the selection criterion discussed before, and for these we run again DFT simulations. Such expanded training set is then used to construct the next generation of SNAP (MLP_1_). Step 1 is then repeated multiple times at a progressively higher MD temperature, which is here increased by 100 K at each step. This process enhances the diversity of the training set and expands the range of temperature at which the SNAP can be used. For MOF-5 we performed iterations until the temperature reached 1000 K, obtaining a total of 487 training configurations. Further details about this approach are described in Supplementary Note 2.

**Fig. 5:**
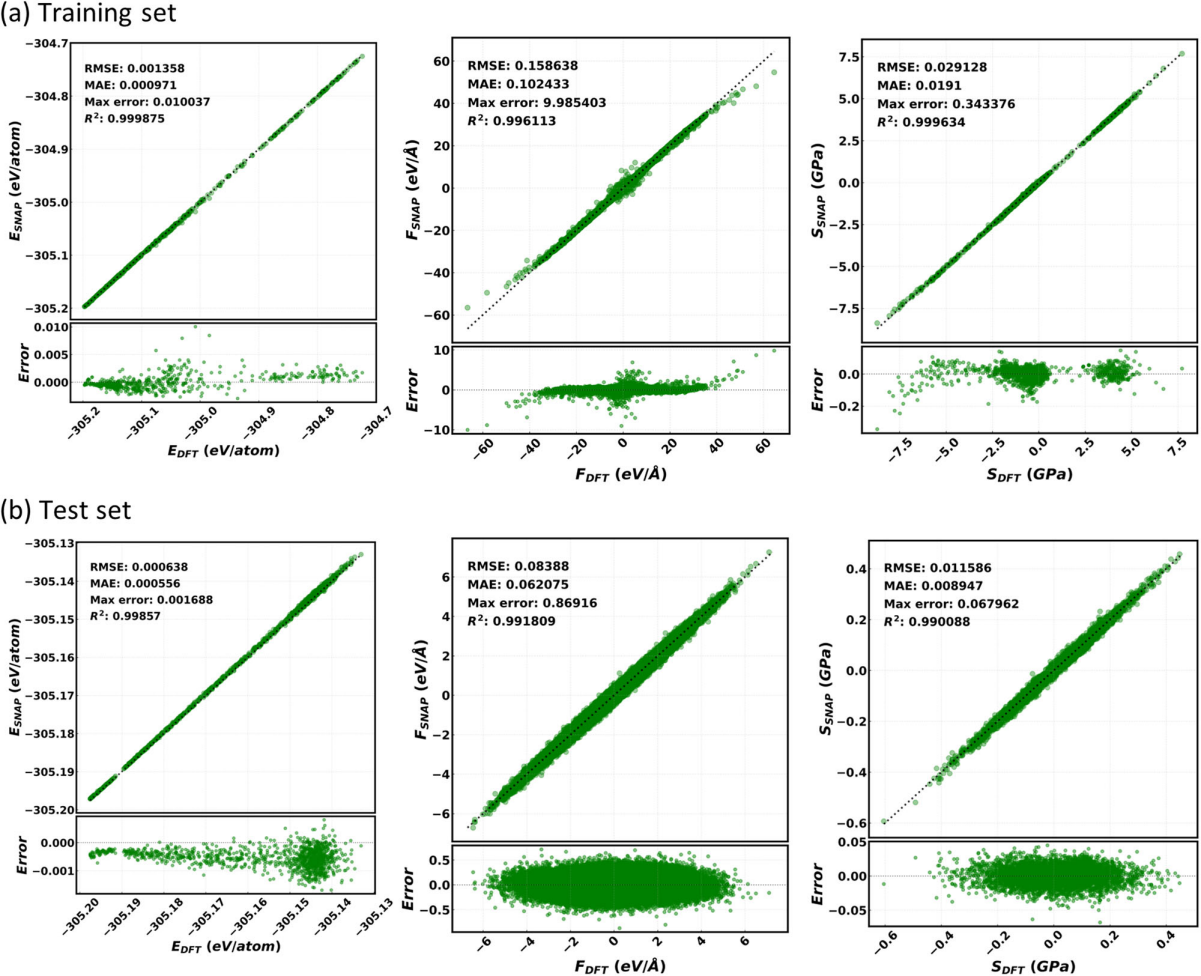
Performance of the trained SNAP model for MOF-5. Parity plots for energy (left-hand side panel), forces (middle panel) and virial-stress components (right-hand side panel) of the MOF-5 SNAP, computed over the training (596 configurations – upper panel) and test set (1191 configurations – lower panels). The RMSE for the test set are 0.6 meV/atom, 84 meV/Å and 11.6 MPa respectively for energy, forces and virial-stress components.

Once the SNAP corresponding to the highest temperature (built over with 487 configurations) is constructed, we perform MD simulations with temperature now ramping between 10 K and 1000 K. In such MD simulations we use the same SNAP, trained over all the 487 configurations, across the entire temperature range and no other previous versions of SNAP are employed. Out of this last MD trajectory, we select the configurations to be used for the test set (1191 in total). Furthermore, we randomly select ~100 more configurations (from this MD simulation and from another at 400 K) to be included in the training set, so that the total number remains close to 600 (596 in our case). More details about the training set construction are given in Supplementary Table 2. The final SNAP is then trained on such data set (with 596 configurations) and used further for all analysis and MD simulations. Parity plots for final training set and test set are shown in Fig. 5. Again, we obtain a very high-quality potential with training-set energies accurate to sub meV/atom, and MAE on forces and stress components of 100 meV/Å and 19 MPa, respectively. Note that the errors on the test set are even lower than those on the training data. This is due to the fact that the test configurations are generated via MD simulations with a properly trained SNAP (where the temperature is ramped between 10 K to 1000 K), and therefore, they are less distorted when compared to the training configurations. As a consequence, the range of values for energy, forces and viral stresses in the test set is more limited than that of the training set. We now proceed to evaluate a number of structural and vibrational properties ZIF-8 and MOF-5 using MD simulations with trained SNAP.

### Lattice constant

**Fig. 6:**
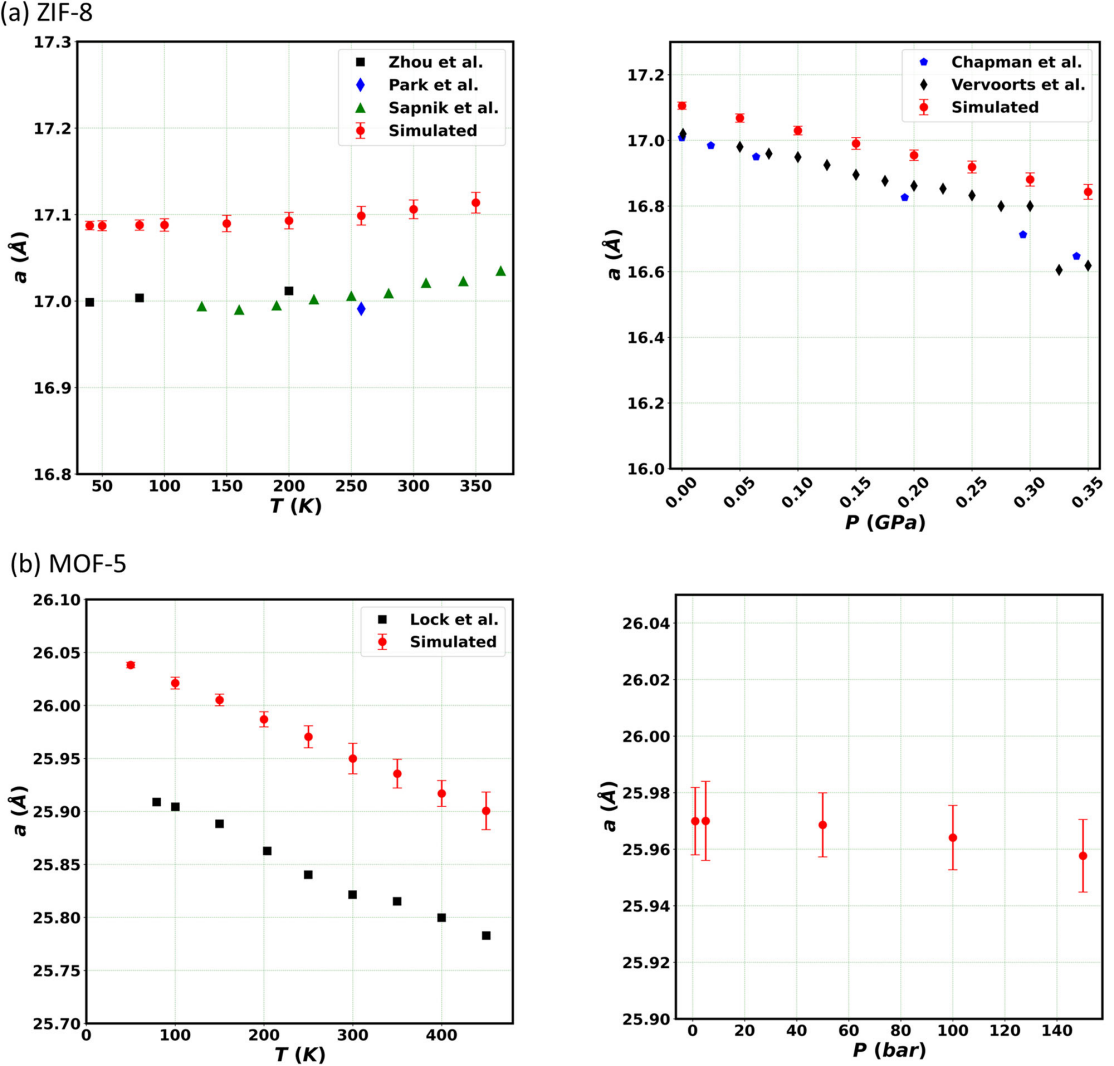
Simulated unit cell parameter for the two MOFs investigated as a function of temperature and pressure. Panel (**a**) is for ZIF-8 and (**b**) for MOF-5, with the results on the left-hand side panels concerning the temperature dependence and those on the right-hand side concerning the pressure. In the various panel we include a comparison with available experimental data (ZIF-8: Zhou et al.^83^, Park et al.^45^, Sapnik et al.^49^, Chapman et al.^84^, and Vervoorts et al.^85^; MOF-5: Lock et al.^52^). For ZIF-8 (MOF-5), the pressure is kept fixed at 1 bar during the temperature scan, while the temperature is kept fixed at 300 K (250 K) during the pressure scan. The error bars over the computed quantities correspond to the variance over the MD trajectory.

We begin by looking at the effect of temperature and pressure on the lattice constant. For this, the trained SNAPs are used to perform five independent 600ps-long MD simulations (with a timestep of 1 fs) in the *NPT* ensemble for both ZIF-8 and MOF-5. Then, the trajectories of the first 100 ps are considered as equilibration steps and the remaining 500 ps are used for property calculations. A window of 10 ps is used to estimate the average and the variance in the lattice parameters, with our results being summarised in Fig. 6. In general, we find an excellent agreement between the simulated lattice parameters and available experimental data. For example, our simulated lattice parameter (26.02 Å) of MOF-5 at 100 K is close to experimental single crystal X-ray diffraction data (25.89 Å) and to the simulation results of Eckhoff et al.^32^ (26.082 Å) and Tayfuroglu et al.^37^ (26.03 Å), obtained with neural-network potentials. In the case of ZIF-8 the lattice parameter increases with temperature (this is referred to as positive thermal expansion) and decreases with pressure. In contrast, MOF-5 has a negative thermal expansion. The computed linear thermal expansion coefficient at 300 K for ZIF-8 is 7.1$\times$10^−6 ^K^−1^, which is within the experimental range determined by 11.9$\times$10^−6 ^K^−1^ (Sapnik et al.^49^) and 6.5$\times$10^−6 ^K^−1^ (Burtch et al.^50,51^). Similarly, we obtain a linear thermal expansion coefficient of −13.3$\times$10^−6 ^K^−1^ for MOF-5 at 300 K, which is close to experimental value^52^ of −13.1$\times$10^−6 ^K^−1^ and to other simulation results from Eckhoff et al.^32^ (−10.5 to −8.3 K^−1^) and Tayfuroglu et al.^37^ (−13.17 to −8.97 K^−1^). Such excellent agreement indicates that our SNAPs are well capable of describing volumetric changes of the lattice parameters as a function of temperature. Note that the absolute value of the lattice parameters predicted by SNAP is slightly larger than that measured experimentally, by approximately 0.5% for both MOFs. This minor overestimation is due to the use of the DFT generalized-gradient approximation (GGA) to the exchange and correlation functional used for the construction of the training set. GGA sometime may slightly underbind and this feature is here transferred to the SNAP.

### Vibrational density of states (VDOS)

**Fig. 7:**
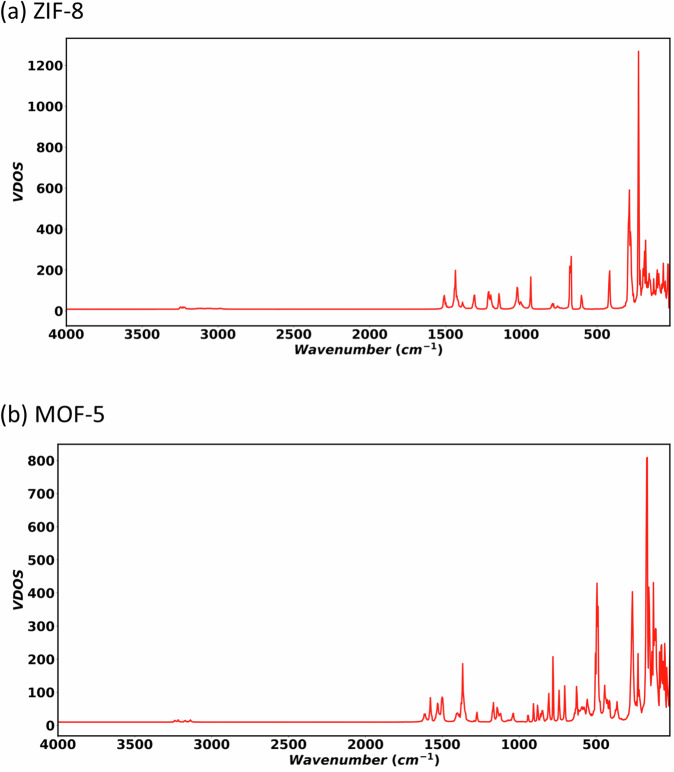
Simulated vibrational density of states (VDOS) for the two MOFs investigated. Panel (**a**) is for ZIF-8 and (**b**) for MOF-5. The corresponding partial VDOSs for each atom types (as defined in Fig. 1) are shown in Supplementary Figs. 11–14.

Having investigated the temperature and pressure response of the MOFs we now move at analysing their vibrational properties. In particular, we compute the vibrational density of states (VDOS), which is here obtained as the Fourier transform of the mass-averaged velocity autocorrelation function along an MD trajectory. In this case, we perform an *NPT* simulation at 300 K for 1 ps, followed by a 500ps-long *NVE* simulation, from which we extract atomic configurations and velocities every 2 fs. Our computed VDOSs are shown in Fig. 7, while the partial VDOS (PVDOS) projected over each atom type are shown in Supplementary Figs. 12 and 14. Similar to results of Eckhoff et al.^32^ for MOF-5, here we observe two main spectral regions (below 1700 cm^−1^ and after 2900 cm^−1^) for both MOF-5 and ZIF-8. Then, we compare the PVDOS (Supplementary Figs. 12 and 14) of different atoms (see Fig. 1 for the definition of the atom types such as C_a_, H_a_, etc.) to identify the modes associated to the various peaks of the vibrational spectrum.

In general, the experimental^53^ infrared (IR) and Raman spectrum of both ZIF-8 and MOF-5 agrees well with our simulated VDOS. Recently, in a detailed computational and experimental study of ZIF-8, Ahmad et al.^54^ identified six regions defining the vibrational spectrum: (i) around 3200 cm^−1^ (stretching modes from C_b_-H_b_), (ii) around 3000 cm^−1^ (C_a_-H_a_ methyl group’s symmetric and asymmetric stretches), (iii) 1400–1500 cm^−1^ (C_a_-H_a_ bending modes, H_b_-C_b_-C_b_-H_b_ rocking modes, and ring deformation modes), (iv) 1310 cm^−1^ (rocking mode of C_b_-H_b_ in the H_b_-C_b_-C_b_-H_b_ moieties, and small deformation of the ring), (v) 1100–1200 cm^−1^ (combined scissoring and rocking motions of C_b_-H_b_ in the H_b_-C_b_-C_b_-H_b_ moieties of different rings in a unit cell, bending modes of C_b_-H_b_ with respect to ring, breathing of entire ring, and minor C_a_-H_a_ bending modes), and (vi) 990 cm^−1^ (C_a_-H_a_ bending modes, in-plane C_b_-H_b_ rocking in the H_b_-C_b_-C_b_-H_b_ moieties, and small in-plane deformation of the ring). Consistently with their study, for the aromatic C_b_-H_b_ dynamics, we observe VDOS spectral amplitude (Fig. 7 and Supplementary Figs. 11–12) in the 3200–3250 cm^−1^ range, which is also close to experimental Raman frequencies^55^ of 3110 and 3131 cm^−1^ and the IR frequency^56^ of 3135 cm^−1^. The simulated VDOS for methyl C_a_-H_a_ dynamics is observed in the window 2900–3150 cm^−1^_,_ which is also in agreement with range of Ahmad et al.^54^ and to the experimental Raman^55^, 2915 and 2931 cm^−1^, and IR frequencies^56^, 2927 and 2961 cm^−1^. We also observe common PVDOS peaks around 1507 and 1140 cm^−1^ for H_b_, C_b_, C_c_, and N atoms, which are associated with the dynamics of the entire ring (N-C_b_, N-C_c_, C_b_-H_b_). This value is close to the experimental Raman frequencies^55^ at 1499 and 1508 cm^−1^. In addition to these, we observe a common peak at 1390 cm^−1^ (for C_a_, H_a_, C_c_, and N atoms), which corresponds to the coupled dynamics of the methyl group and the ring. For all C, H, and N atoms common peaks are observed near 1400–1450, 1310, 1200, 1000–1050, and 650–700 cm^−1^, which correspond to vibrational dynamics of entire organic segment of ZIF-8. A peak around 600 cm^−1^ is common to H_b_, C_b_, and N atoms and corresponds to associated bending modes. Further analysis of PVDOS reveals Zn-N vibrational frequencies at around 180 cm^−1^, 226 cm^−1^ and 286 cm^−1^, which are close to the experimental Zn-N Raman frequencies^55^ of 168 and 273 cm^−1^ and the far infrared (IR) frequencies^57^ in the 265–325 cm^−1^ range. We also observe various peaks below 300 cm^−1^ which corresponds to collective atomic vibrations of ZIF-8.

Moving to MOF-5, we observe phonon bands up to 1650 cm^−1^ in the first spectral region and after 3000 cm^−1^ in the second spectral region. According to experimental IR/Raman spectra, Civalleri et al.^58^ defined five spectral regions for MOF-5: (i) 2900–3100 cm^−1^ (due to C-H stretching in phenylene), (ii) 1300–1650 cm^−1^ (carboxylate C=O and phenylene C=C stretching, and C-H bending vibrations), (iii) 600–1200 cm^−1^ (in-plane and out-of-plane deformation of phenylene ring including C-H groups), (iv) 200–600 cm^−1^ (due to Zn-O stretching and bending), (v) below 200 cm^−1^ (due to collective atomic vibrations and lattice modes). The PVDOS (Supplementary Figs. 13–14) reveals C_c_-H stretching frequencies between 3050–3250 cm^−1^, which are slightly outside the 2900–3100 cm^−1^ range, but are consistent with the frequencies obtained with the neural-network potentials simulations of Tayfuroglu et al.^37^ (3126.4 and 3138.9 cm^−1^) and Eckhoff et al. (3104/3148 cm^−1^). In the PVDOS between 1250–1650 cm^−1^ we observe several peaks corresponding to carboxylate C=O, phenylene C=C, and C_c_-H vibrations. These are in good agreement with experimental^59^ C-O vibrational frequencies, 1377 and 1585 cm^−1^, and previous simulation^37^ results. Furthermore, we observe vibrational frequencies at 486–493 cm^−1^ and 556 cm^−1^ for O_4_-Zn and frequencies in the 426–443 cm^−1^ range for O_2_-Zn, which are close to the experimental^53,59^ Zn-O IR frequency at 523 cm^−1^. We also observe lower O_2_-Zn frequencies at 263 cm^−1^ and 363 cm^−1^, which are consistent with above mentioned spectral regions of the Zn-O stretching and bending modes. We further find various peaks below 200 cm^−1^, which are attributed to collective atomic vibrations including both the metal nodes and organic linkers.

### Free energy barrier for rotation of the MOF-5 phenylene rings

In order to unravel the internal dynamics of a MOF, it is essential to develop an understanding of the free energy barriers for the internal dynamics of different groups^4,60–63^. Such barriers can be studied with our MLP, which should be able to reliably map the atomic environments in the transition-state zone of the phase space. In MOF-5 the phenylene rings do not have a significant steric hinderance, however, significant interaction with the neighboring atoms creates a barrier to their rotational dynamics along the central axis. Therefore, in our MD simulations for MOF-5 at room temperature we did not observed rotation of any phenylene ring.

**Fig. 8:**
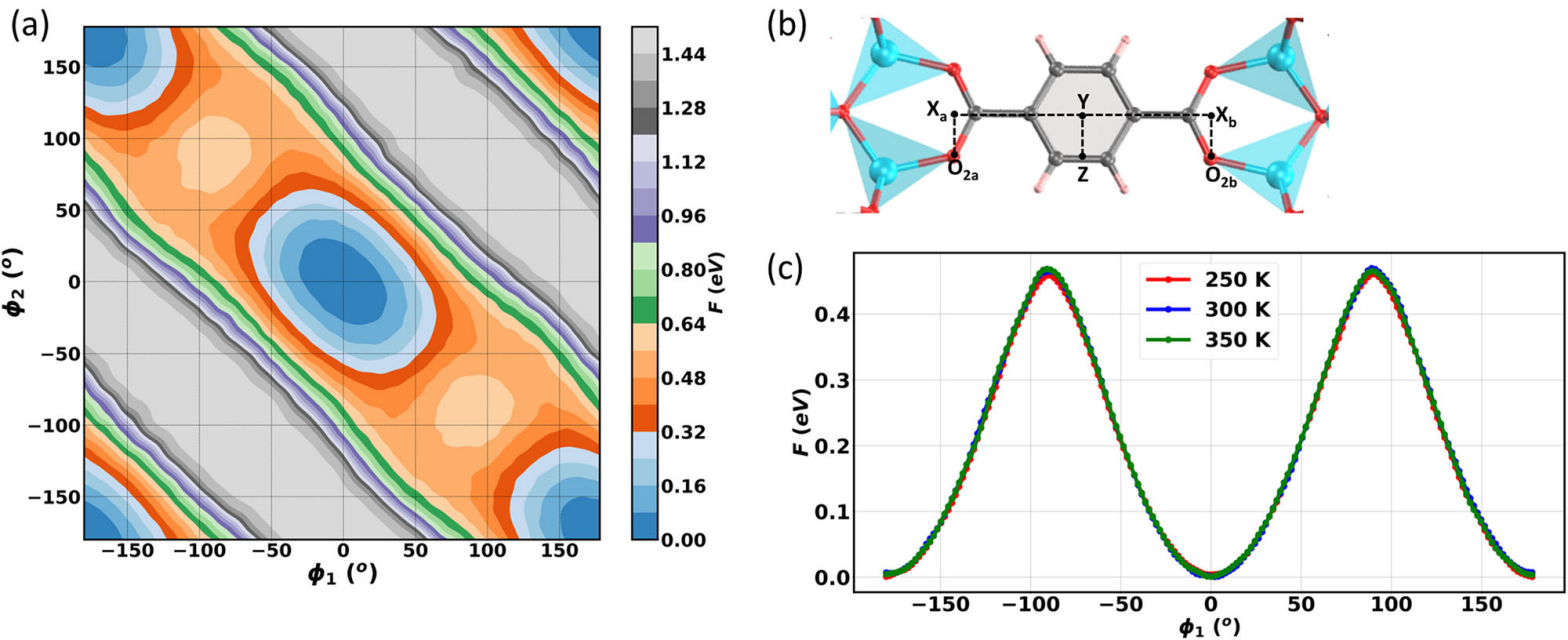
Investigation of the MOF-5 phenylene ring rotation performed with the trained SNAP. **a** Free energy profile for rotation of a phenylene ring in MOF-5 as a function of the two dihedral angle collective variables ${\phi }_{1}$ and ${\phi }_{2}$. The free energy minimum is shown in blue, and the barriers is shown in orange. **b** Schematic showing the collective variables, ${\phi }_{1}={\phi }_{{O}_{2a}{X}_{a}{YZ}}$ and ${\phi }_{2}{=\phi }_{{O}_{2b}{X}_{b}{YZ}}$. **c** Free energy for the phenylene ring rotation as a function of one of the collective variables at different temperatures. A rotational barrier of 0.46 eV is observed for rotation of a phenylene ring in MOF-5.

We have then analysed the distribution of the dihedral angles in MOF-5 (see Supplementary Fig. 9) in the training set configurations (generated with our temperature-driven active leaning algorithm at temperatures comprised between 100–1000 K). We have found that the training set contains configurations with all possible values of the C_c_-C_b_-C_a_-O_2_ dihedral angles (−180^o^ to 180^o^). Thus, with our approach, the trained SNAP can reliably map atomic environments near the transition state corresponding to the rotational barrier. This motivates us to quantify the free-energy barrier for phenylene ring rotation in MOF-5. Earlier simulation work returned an energy barrier of 0.508/0.491 eV^32^ and 0.58/0.65 eV^37^ for the rotation of the phenylene ring in MOF-5, although these studies did not consider entropy effects. In order to evaluate the free-energy barrier (which includes both energy and entropy contributions) for the rotation of the phenylene ring, here we perform well-tempered metadynamics (WTM) simulations. In the WTM calculation, we consider two dihedral angles (${\phi }_{1}$ and ${\phi }_{2}$) at each side of the phenylene ring as collective variables (see Fig. 8). All the WTM simulations are performed in the *NVT* ensemble, therefore, the resultant free energy corresponds to the Helmholtz free energy. Additional details about the WTM simulation are given in Methods section and Supplementary Note 3.

The free energy profile as a function of both collective variables is shown in Fig. 8a. The stable states in the rotation (blue regions) are separated by transition states (orange regions). If we consider the simplified case where the MOF-5 structure is rigid and only the rotational motion of the phenylene rings is allowed, one will have a negative correlation between the two collective variables and the only free-energy variation will be on the diagonal line of the 2D mesh of Fig. 8a. Along this diagonal, we compute a free energy barrier of more than 0.6 eV. Since the MOF-5 structure is flexible, the oxygen atoms wobble with respect to the Zn ones, a feature that relaxes the negative correlation between the collective variables and makes the free energy profile broader. This wobbling results in a transition path presenting a lower free-energy barrier than that along diagonal path, hence the wobbling of oxygen atoms helps in the rotation of phenylene rings of MOF-5 (see Supplementary Movie 1). The free-energy profile as a function of one dihedral angle, Fig. 8c, is finally obtained by integrating the effect of other angle^4^ and a rotation free-energy barrier of 0.46 eV is thus computed. This value is close to the experimental one^63^ of 0.49 eV, indicating once again the excellent quality of our interatomic potential.

## Discussion

In the last few years, the application of MLPs to the study of MOFs has received a growing attention. In past studies, to select the training set configurations for the development of MLPs, two types of approaches have been reported. The first one involves the use of a configuration-selection metric, such as the model deviation (e.g., DP-GEN^64^) or the uncertainty quantification^26,65^, to decide upon the inclusion of a configuration in the training set. Another approach uses biased simulation (e.g., metadynamics) and selects configurations separated by 1 ps simulation time to avoid correlation and to ensure diversity, without considering any particular configuration selection metric^36^. Here we focus primarily on bonded atomic systems (such as MOFs, small molecules, etc.). Therefore, our active learning algorithm relies on internal coordinates (bonds, angles, dihedrals), and cell parameters (for periodic configurations) and uses these as a configuration selection metric. The distribution of these values also gives an idea about the diversity of the training set. We avoid correlation and ensure diversity by selecting configurations from short (around 1 ps long) MD simulations (starting with different initial structure and velocities) at increasing temperatures. In our approach, we have not used any biased simulations, where the configuration space unravels along only a few collective variables. Instead, we vary the temperature, a strategy that allows the configuration space to expand in all possible directions. Geometries selected from this approach contribute to develop an effective MLP, which allows us to explore the configuration space in the direction of any feasible collective variables (as shown for the rotation of phenylene ring) and study the relevant transition states.

The complexity of the MLP training process can be understood by considering a typical MOF with *N*_*a*_ atoms in the unit cell. The DFT calculation of energy, forces and stress of *N*_*c*_ configurations will result in (3*N_*a*_ + 7)*N_*c*_ data points. This means that for a MOF with 400 atoms in the unit cell and 500–1000 configurations, there will be around 6–12 $\times$10^5^ data points. Then the data are used to fit the MLP model. Here, we have used SNAP, a MLP linear model constructed over only a few hundreds parameters (392 in our case). The training of a few hundreds parameters on such a large training set can be performed just on a laptop in a few minutes. This contrasts the training process of neural-network potential models, such as NequIP^36,66^ and MACE^67,68^, which requires the determination of a large number of parameters (of the order of 10^5^–10^6^) and it is usually performed on high-memory graphical-processing units in a time comprised between a few hours to a day. Having a large number of parameters, these deep-learning MLP models require more extended training data sets, but they are typically more accurate (e.g., force error ~ 30 meV/Å) than SNAP (force error ~ 60 meV/Å). The typical inference times are then more difficult to compare. In general, since linear models can be considered as a single-layer neural network, they are quicker to run. However, the final running time depends strongly on the time required to calculate the structure descriptors, which can vary widely depending on the specific implementations. In our MD simulations, we obtained speed of 0.35 s per MD step (with SNAP and D3 corrections) on a single core. Finally, as we have demonstrated here, our approach is general and can be widely deployed to construct high-performing MLPs at a low computational cost to accurately study the internal dynamics of MOFs. It is likely that the same SNAP is not able to describe bond-breaking events (at extreme temperature and pressure conditions) and other phenomena involving chemical reactions, such as adsorption and catalysis. This, however, is not an intrinsic limitation of SNAP, as of deep-learning MLPs, but rather depends on the specific configurations included in the training set. In the future, we will explore the use of SNAP for the study of such phenomena, including diffusion of gas molecules^34,42^ in MOFs and chemical reactions.

## Methods

### Density functional theory (DFT) calculations

The QUICKSTEP^69^ module of the CP2K^70^ package is used for all the DFT calculations. Within this approach, the Kohn–Sham molecular orbitals are expanded over a linear combination of atom-centered Gaussian-type orbitals. All atoms are described using the MOLOPT basis set in combination with norm-conserving Goedecker–Teter–Hutter^71^ (GTH) pseudopotentials. The Perdew–Burke–Ernzerhof (PBE)^72^ exchange-correlation functional is employed throughout and the electron density is written over an auxiliary plane-wave basis set with appropriate energy cut-off (different for different types of calculations). The orbital transformation approach is used to find the solution of the Kohn–Sham equations and the self-consistent field (SCF) convergence of both the outer and inner loops is achieved with an accuracy of 10^−7^ Hartree.

Here, DFT is used for calculating energy, forces, and stress tensor values for a given atomic configuration, data that are used to construct the SNAP. In these calculations, DFT-D3^73^ corrections are not included, and an energy cut-off of 1000 Ry is used. In addition, DFT is also employed to perform geometry and cell optimization of both ZIF-8 and MOF-5. In this case, dispersion corrections are included using the DFT-D3^73^ approach with Becke-Johnson (BJ) damping^74^ and an energy cut-off of 1000 Ry.

### Ab-initio molecular dynamics simulations (AIMD) of ZIF-8

In order to generate the different atomic configurations of ZIF-8, AIMD simulations are performed. We first optimize the ZIF-8 unit cell (containing 276 atoms) using DFT with an energy cut-off of 600 Ry. Then, we perform CP2K AIMD simulations at different temperatures (100 to 1000 K in intervals of 100 K). The AIMD simulations are performed at constant temperature and pressure using a flexible cell. A timestep of 0.5 fs is used and all AIMD simulations are performed for 2000 steps (namely for 1 ps) at each temperature. To maintain the temperature, the Nose-Hoover thermostat is employed with time constant of 25 fs. The pressure is kept at 1 bar with the help of a barostat (as implemented in CP2K) with a time constant of 50 fs.

### Molecular dynamics simulations

The trained SNAP^43^ is used to perform molecular dynamics (MD) simulations of both ZIF-8 and MOF-5 using the LAMMPS^47^ package. In addition to the SNAP, dispersion corrections are also included in these MD simulations using the Grimme’s D3^73–75^ approach with BJ damping. Namely, at each MD step, D3 corrections (to energy, forces, and virial-stress values) are added to the corresponding estimates from SNAP. Then, the atomic positions, velocities, and cell parameters are updated accordingly. A timestep of 1 fs is used in all LAMMPS MD simulations. For ZIF-8, an additional repulsive Ziegler-Biersack-Littmark (ZBL)^76^ empirical potential is employed to create repulsion between the H_b_-H_a_, H_b_-N, H_b_-Zn, H_a_-C_b_, H_a_-N, and H_a_-Zn atom pairs. The inner and outer cut-off radius of the ZBL potential are chosen at 1.8 Å and 2.3 Å, respectively. For the atomic configurations contained in the training set of ZIF-8 the considered atomic pairs have a distance longer than 2.5 Å, resulting in zero ZBL contribution to the energy, forces, and stress. Therefore, the effect of ZBL is not subtracted from the training set data. In the case of MOF-5, no ZBL repulsion is considered.

The performance of SNAP against the lattice constants is estimated through MD simulations in the isothermal-isobaric ensemble, with constant number of particles, *N*, constant pressure, *P*, and temperature, *T*. In these MD simulations, a Nose-Hoover thermostat with 5 chains and time constant of 100 fs is used to maintain the temperature and a Nose-Hoover barostat (with time constant of 200 fs) balances the pressure. During these simulations, only the cell parameters are allowed to change, while the cell angles are kept constant.

### Well-tempered metadynamics simulations

We perform well-tempered metadynamics (WTM)^77,78^ simulation using the open-source community-developed Plumed^79,80^ library patched with LAMMPS^47^. In the WTM simulations, a bias potential is added along the collective variables (CVs), to probe the free-energy landscape. In this work, we use two dihedral angles (described in main manuscript) as CVs. In the WTM simulations, we use a Gaussian width of 0.1 radian, an initial gaussian height of 0.05 eV, a biasfactor of 12, a grid spacing of 0.05 radian, and a bias deposition rate of 10^4 ^ns^−1^ (every 100 simulation steps). To maintain stability in the WTM simulations, we apply upper walls on four Zn-O distances (near to the considered phenylene ring) at a value of 2.4 Å with force constant of 25 eV-Å^2^. In addition to these, upper and lower walls are applied to two O_2_-Zn-Zn-O_2_ dihedral angles at a value of 0.85 radian with force constant of 25 eV-radian^2^. We have performed WTM simulations at different temperatures in the isothermal ensemble.

## Supplementary information

Supplementary Information

Supplementary Video 1

## Data Availability

All datasets developed and used in this work are available via Zenodo^81^.

## Code availability

Our Python library and few examples of its use for training the SNAP and other different steps of temperature drive active learning are available at Github^82^ in MOF_MLP_2024 repository.
