# Supplementary material for: Quantum-accurate machine learning potentials for metal-organic frameworks using temperature driven active learning
Source: NPJ Comput Mater. 2024 Oct 8;10(1):237. doi: 10.1038/s41524-024-01427-y (PMC11461275; doi:10.1038/s41524-024-01427-y)
Supplement: Supplementary file 1 — Supplementary Information [file 41524_2024_1427_MOESM1_ESM.pdf]

# Supplementary Information

## Quantum-Accurate Machine Learning Potentials for Metal-Organic Frameworks using Temperature Driven Active Learning

Abhishek Sharma[*] and Stefano Sanvito[*]

[*]School of Physics, AMBER and CRANN Institute, Trinity College, Dublin 2, Ireland.

Corresponding author emails: asharma.ms.in@gmail.com (A.S.), sanvitos@tcd.ie (S.S.)

## Supplementary Note 1: CBAD algorithm

Pseudocode corresponding to CBAD algorithm is given in Supplementary Figure 1 and a python library *dam* implementing this pseudocode is available on GitHub.[1] Details about size of training sets obtained from trajectory of *ab-initio* molecular dynamics (AIMD) simulations using different values of descriptor resolutions are given in Supplementary Table 1.

```
Δcl = 0.0; Δca = 0.0; Δb = 0.0; Δa = 0.0; Δd = 0.0;

Cr = [[],[],[],[],[],[]]; # for a, b, c, α, β, γ
Br = [[],[],[],…,[]];    # for l bond types
Ar = [[],[],[],…,[]];    # for m angle types
Dr = [[],[],[],…,[]];    # for n dihedral types

for i = 1 to N_C    # N_C number of configurations
{    check = 0;
     S = get_CBAD(i); #This function will return CBAD values of a configuration

for j = 1 to 3    # for a, b, c
    { if(int(S. C[j]/Δcl) not in Cr[j] ) { check = check + 1; } }
for j = 4 to 6   # for α, β, γ
    { if( int(S. C[j]/Δca) not in Cr[j] ) { check = check + 1; } }

for j = 1 to l    # l bond types
   {  for k = 1 to B_j    # B_j number of bonds of type j
        { if(int(S. b^j_k/Δb) not in Br[j] ) { check = check + 1; } } }

for j = 1 to m   # m angle types
   {  for k = 1 to A_j    # A_j number of angles of type j
        { if(int(S. a^j_k/Δa) not in Ar[j] ) { check = check + 1; } } }

for j = 1 to n   # n dihedral types
   {  for k = 1 to D_j    # D_j number of dihedrals of type j
        { if(int(S. d^j_k/Δd) not in Dr[j] ) { check = check + 1; } } }

     if(check== 6+nbonds + angles + dihedrals)
     { include_in_training_set(S);
       update(Cr,Br,Ar,Dr,S);
     }
}
```

**Supplementary Figure 1:** A pseudocode illustrating CBAD active learning algorithm for deciding which configuration to include in the training set. This algorithm compares cell-parameters, bonds, angles, and dihedrals (CBAD) of a given configuration with the existing values of the training set and includes the configuration in training set if either of the values is not included in the training set. A python code implementing this pseudocode is available on GitHub.[1]

**Supplementary Table 1:** Details about size of different training sets obtained from trajectory of *ab-initio* molecular dynamics (AIMD) simulations using different values of descriptor resolutions (main parameters of CBAD algorithm) for cell lengths ($\Delta_{cl}$), cell angles ($\Delta_{ca}$), bond lengths ($\Delta_{b}$), bond angles ($\Delta_{a}$), and dihedral angles ($\Delta_{d}$).

| **S.No.** | **Descriptor Resolutions** | | | | | **Training Set Size** |
|---|---|---|---|---|---|---|
| | $\Delta_{cl}$ (Å) | $\Delta_{ca}$ (°) | $\Delta_{b}$ (Å) | $\Delta_{a}$ (°) | $\Delta_{d}$ (°) | |
| 1 | 2.0 | 64.0 | 1.0 | 50.0 | 100.0 | 45 |
| 2 | 1.0 | 32.0 | 0.5 | 30.0 | 60.0 | 68 |
| 3 | 0.5 | 16.0 | 0.5 | 20.0 | 40.0 | 104 |
| 4 | 0.2 | 8.0 | 0.2 | 10.0 | 20.0 | 227 |
| 5 | 0.05 | 4.0 | 0.05 | 5.0 | 10.0 | 489 |
| 6 | 0.02 | 4.0 | 0.05 | 5.0 | 10.0 | 609 |
| 7 | 0.02 | 4.0 | 0.04 | 4.0 | 8.0 | 672 |
| 8 | 0.01 | 2.0 | 0.02 | 2.0 | 4.0 | 1230 |
| 9 | 0.005 | 1.0 | 0.01 | 1.0 | 2.0 | 2132 |

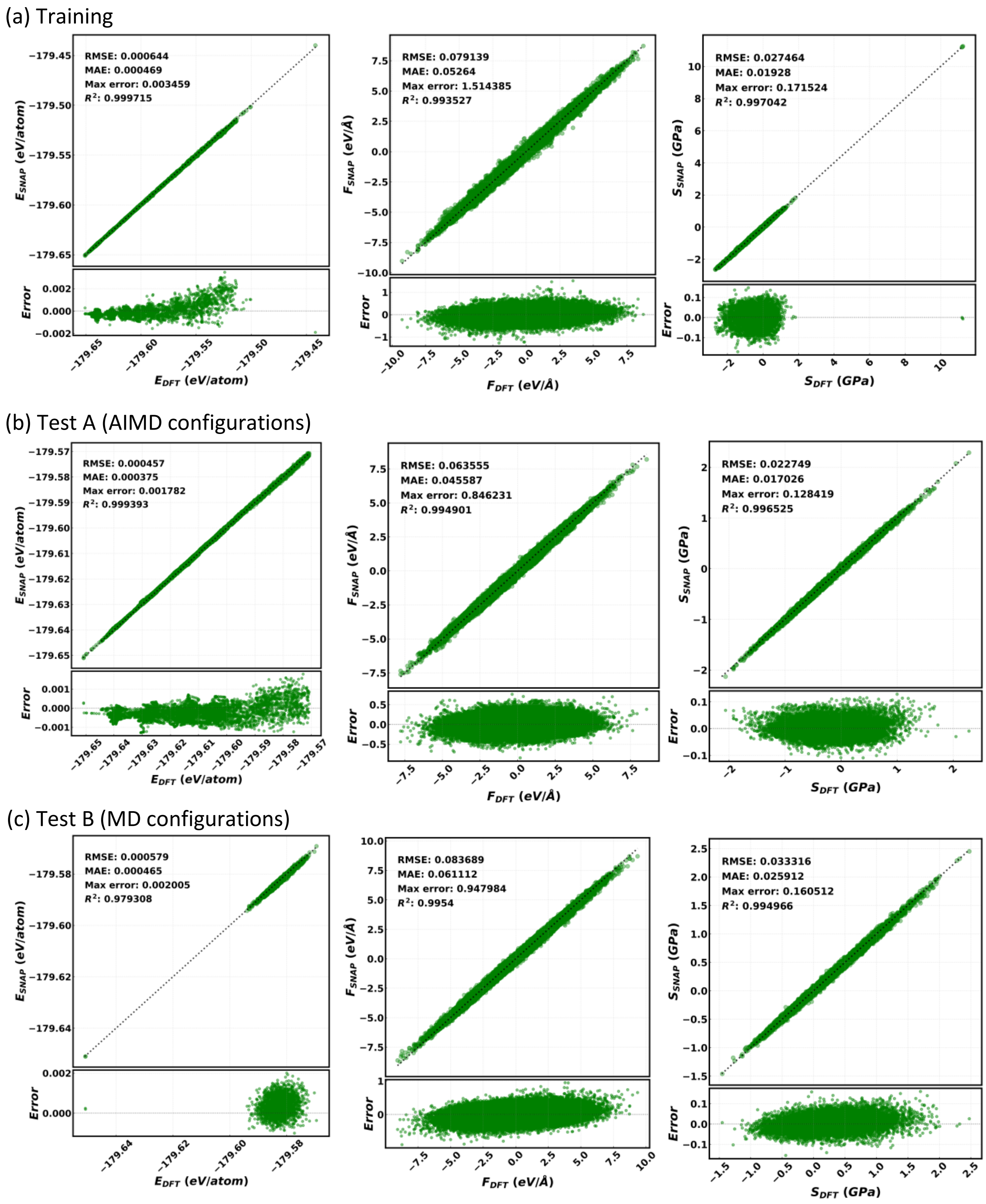


**Supplementary Figure 2:** Parity plots of energy (E), forces (F), and stress (S) values for (a) training set (3138 configurations), (b) test set A (4956 configurations selected from *ab-initio* molecular dynamics (AIMD) simulations), and (c) test set B (2379 configurations selected from classical molecular dynamics (MD) simulation) of ZIF-8. These parity plots compare energy (E), forces (F), and virial-stress (S) values of different configurations of ZIF-8, obtained using spectral neighbor analysis potential (SNAP) and density functional theory (DFT). Values of root-mean square error (RMSE), mean absolute error (MAE), and maximum error are given in each parity plot.

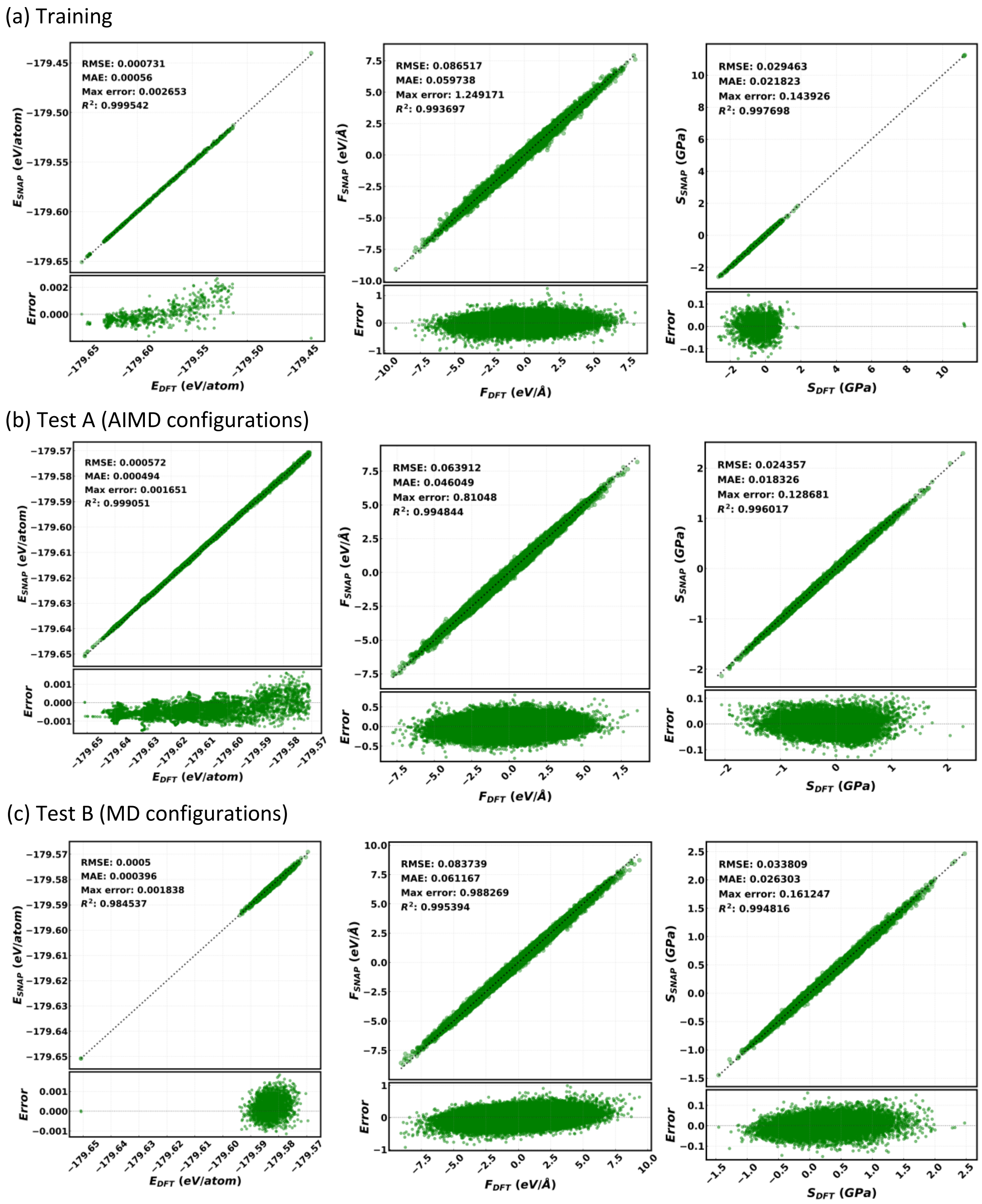


**Supplementary Figure 3:** Parity plots of energy (E), forces (F), and stress (S) values for (a) training set (672 configurations), (b) test set A (4956 configurations), and (c) test set B (2379 configurations) of ZIF-8. These parity plots compare energy, forces, and virial-stress values of different configurations of ZIF-8, obtained using SNAP (trained over 672 configurations) and DFT.

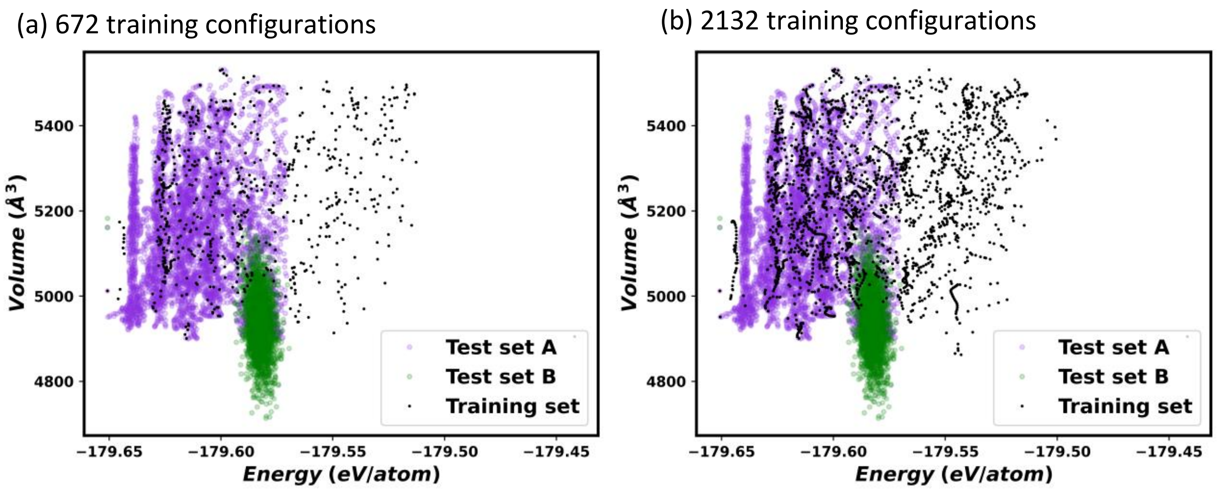


**Supplementary Figure 4:** Distribution of energy and volume for test set A (4956 configurations), test set B (2379 configurations), and training set with (a) 672 and (b) 2132 configurations of ZIF-8. The training set configurations and test set A configurations are selected from trajectory of AIMD simulations at different temperatures (for the duration of 1 ps with timestep of 0.5 fs) in the range of 100-1000 K and 100-500 K, respectively. The configurations in the 1 ps AIMD simulation trajectory are correlated (as observed in distribution of points along a curved line in test set A), therefore configurations in the bigger training set as shown in (b) could also have some degree of correlation.

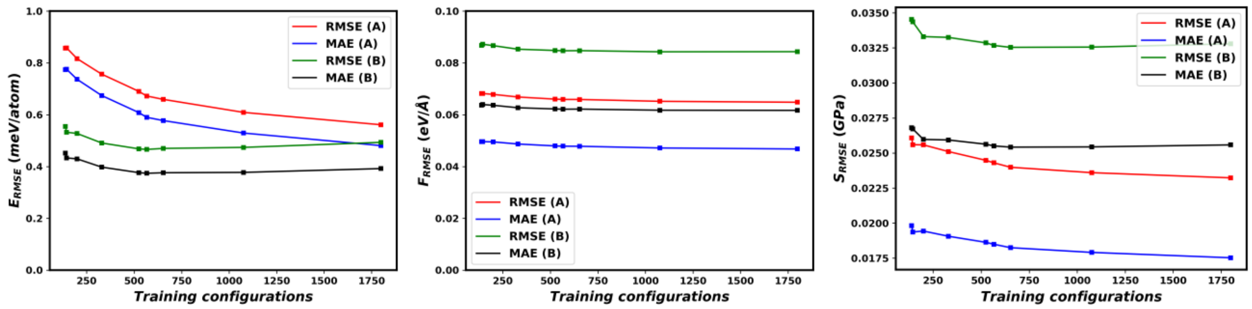


**Supplementary Figure 5**: Learning curves for the RMSE and MAE for energy (left-hand side panel), forces (middle panel) and virial stress (right-hand side panel) values of ZIF-8. Here the training configurations are selected from shuffled AIMD simulation trajectories and data are presented for test set A (composed of 4956 configurations from AIMD simulations) and test set B (composed of 2379 configurations from classical molecular dynamics (MD) simulations) as a function of the number of configurations in the new training set.

## Supplementary Note 2: Implementation of Temperature Driven Active Learning Algorithm for MOF-5

In order to generate the training set configurations of MOF-5, we used our temperature driven active-learning algorithm. This algorithm starts with the experimental structure and uses the previously discussed CBAD algorithm. In addition to Figure 4 of main manuscript, details about the steps taken to generate the training set of MOF-5 are given in Supplementary Table 2 and described below.

**Supplementary Table 2:** Details of the steps involved in the temperature-driven active-learning algorithm to obtain the training set configurations for MOF-5.

| Step | Description | Training Set Size | Resulting MLP |
|---|---|---|---|
| Step0 | Starting with the experimental structure, 200 configurations are generated using random perturbations of the atomic positions. Out of these 200 configurations, 50 configurations are selected using the CBAD algorithm for the training set and DFT calculations are performed for these 50 selected configurations. | 51 (50 + 1 experimental structure) | $MLP_0$ |
| Step1 | With the trained $MLP_0$, 50 separate MD simulations are performed (starting with different initial structures and velocities) at 100 K for 1 ps. From each MD trajectory, at most one configuration is selected using the CBAD algorithm and a DFT calculation is performed for each selected configuration. | 100 (49 configurations selected in this step + 51 old configurations) | $MLP_1$ |
| Step2 | With the trained $MLP_1$, 50 separate MD simulations are performed (starting with different initial structures and velocities) at 200 K for 1 ps. From each MD trajectory, at most one configuration is selected using the CBAD algorithm and a DFT calculation is performed for each selected configuration. | 147 (47 configurations selected in this step + 100 old configurations) | $MLP_2$ |
| Step3 | With the trained $MLP_2$, 50 separate MD simulations are performed (starting with different initial structures and velocities) at 300 K for 1 ps. From each MD trajectory, at most one configuration is selected using the CBAD algorithm and a DFT calculation is performed for each selected configuration. | 195 (48 configurations selected in this step + 147 old configurations) | $MLP_3$ |
| ⋮ | ⋮ | ⋮ | ⋮ |
| Step10 | With the trained $MLP_9$, 50 separate MD simulations are performed (starting with different initial structure and velocities) at 1000 K for 1 ps. From each MD trajectory, at most one configuration is selected using the CBAD algorithm and a DFT calculation is performed for each selected configuration. | 487 (47 configurations selected in this step + 440 old configurations) | *$MLP_{10}$ |

| | | | |
|---|---|---|---|
| Step11 | With the trained $MLP_{10}$, one MD simulation is performed 400 K. From this MD trajectory, a few configurations are selected randomly with minimum gap of 10 ps and a DFT calculation is performed for each selected configuration. | 537 (50 configurations selected in this step + 487 old configurations) | |
| Step12 | With the trained $MLP_{10}$, one MD simulation is performed with temperature ramping between 10 to 500 K. From this MD trajectory, a few configurations are selected randomly with minimum gap of 10 ps and a DFT calculation is performed for each selected configuration. | 596 (59 configurations selected in this step + 537 old configurations) | *$MLP_{11}$ |

***In this work, only to obtain test set configurations and a few training set configuration the $MLP_{10}$ (trained over 487 configurations) was employed. For all the property calculations via MD simulations of MOF-5, the final version of MLP (i.e., $MLP_{11}$ trained over 596 configurations) is used.**

In the experimentally obtained MOF atomic structure, the position of the hydrogen atoms is obtained using the riding model.[2,3] Compared to DFT-based equilibrium C-H bond lengths (> 1.0 Å), the riding model-based C-H distances (0.95/0.98 Å) are shorter. Therefore, the DFT calculation of the experimental structure results in a higher energy. In Step0 of our algorithm, we start with the experimental structure and create different configurations using random perturbations of the atomic positions. Therefore, the energy of the selected configurations (using the CBAD algorithm) in Step0 are higher (primarily due to small C-H distances) compared to configurations obtained in other steps. The DFT energies of the training configurations obtained at different temperature-driven steps of our algorithm are shown in Supplementary Figure 6.

In Step1, we perform MD simulations (at the lower temperature of 100 K) using $MLP_0$ (trained over configurations selected in Step0) and select configurations for the training set from the MD trajectories. The $MLP_0$, is an initial approximation of potential energy surface, therefore, we first perform MD simulations at low temperature to avoid creating atomic configurations with unphysical distortions. The training set obtained in Step1, has many high energy configurations (due to least accurate $MLP_0$) and a few low energy configurations too. Then, in each step (Step2 to Step10), we increase the temperature by 100 K (200 K to 1000 K), perform MD simulations to generate a diverse set of configurations and select configurations using the CBAD algorithm. In each successive step, we obtain configurations with higher energy and more structural diversity (see Supplementary Figure 6, 9 and 10 for details), and training over these results in a better MLP. After Step10 (MD simulation at 1000 K), to make the training set size close to 600 configurations, we add a few configurations in the training set from the MD simulation at 400 K (Step 11) and the MD simulation with temperature ramping between 10 to 500 K (Step 12). For further details about different steps, see Supplementary Table 2.

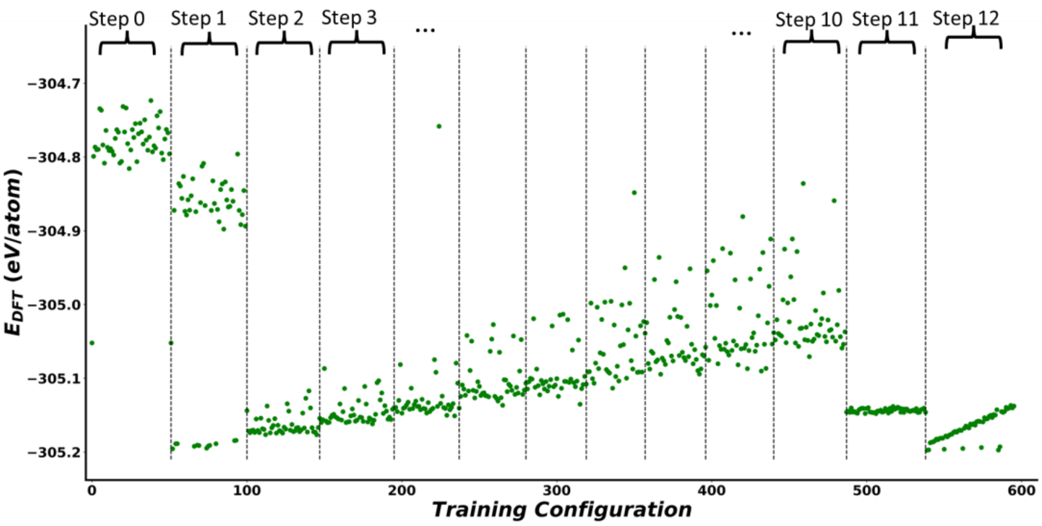


**Supplementary Figure 6:** Energy of the MOF-5 training set configurations (obtained using DFT) obtained at different temperature-driven active-learning steps (see Supplementary Note 2 and Supplementary Table 2 for details about the steps).

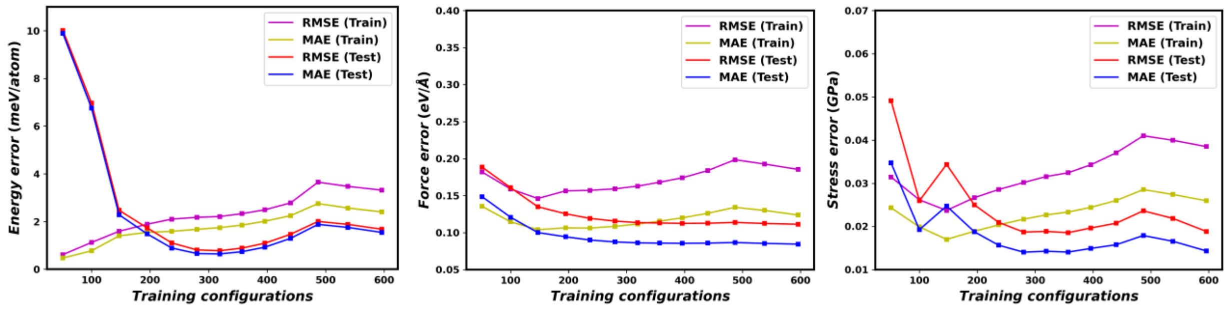


**Supplementary Figure 7**: Learning curves for the RMSE and MAE for energy (left-hand side panel), forces (middle panel) and virial stress (right-hand side panel) of MOF-5. Here the training configurations are generated using different steps of the temperature-driven active-learning algorithm. An increment in errors in energy and virial stress after 300 configurations is observed due to an increase in the diversity of the configurations. After training set reaches 500 configurations, further training configurations are included from a MD simulation trajectory, which have lesser diversity, therefore a decrease in the errors is observed.

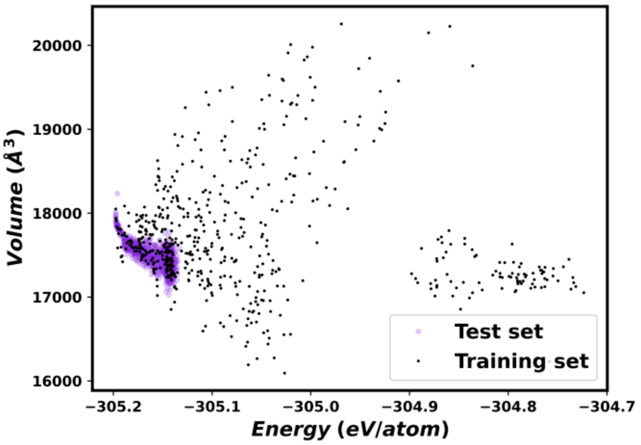


**Supplementary Figure 8:** Distribution of energy and volume for test set (1191 configurations) and training set (596 configurations) of MOF-5. Here the training set is generated using temperature driven active learning algorithm which shows no sign of correlation.

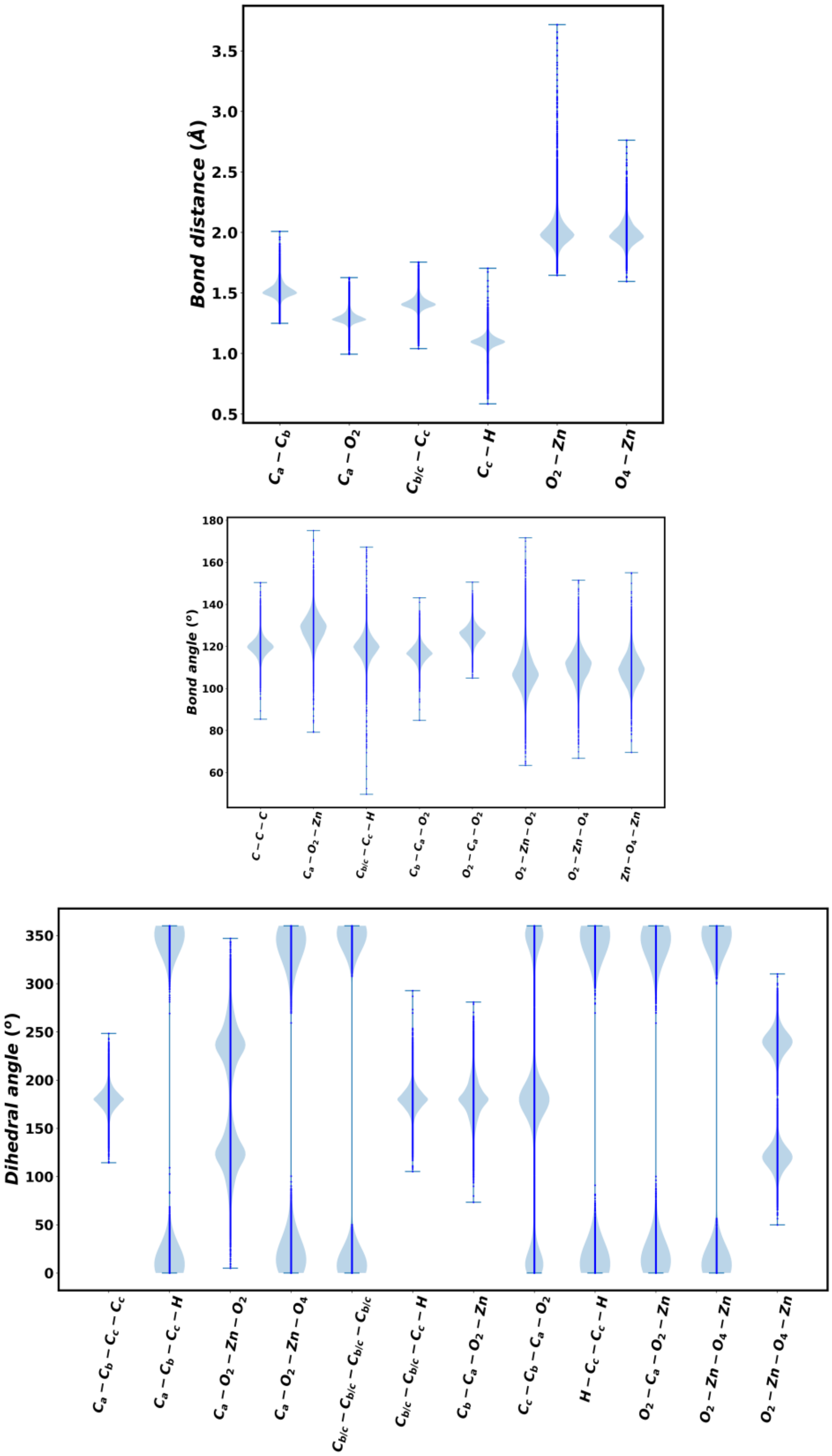


**Supplementary Figure 9:** Distribution of bond distance, bond angles, and dihedral angles in final MOF-5 training set configurations (with 596 configurations).

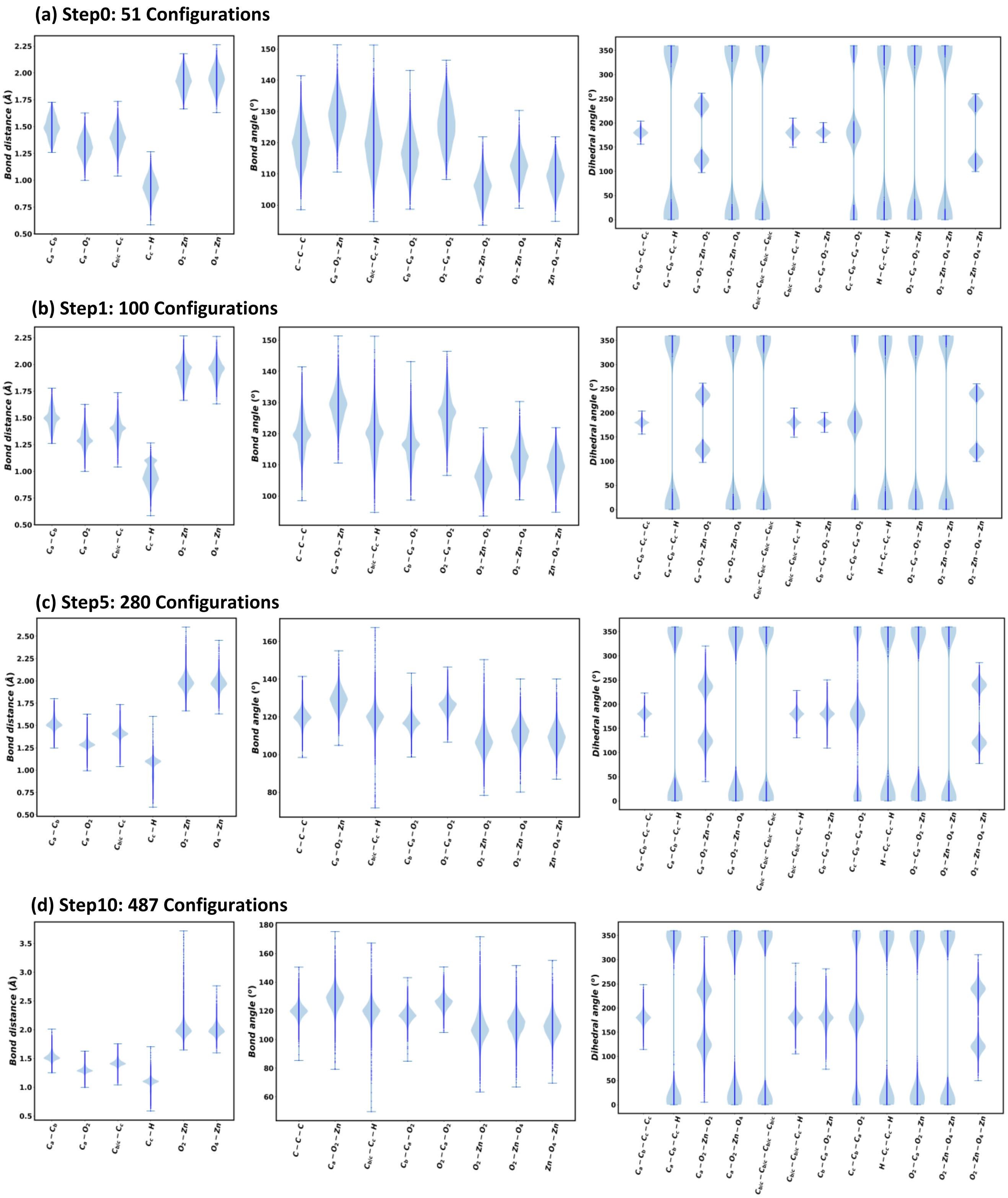


**Supplementary Figure 10:** Variation of the structural diversity (i.e., bond distance, bond angles, and dihedral angles) in the training set at different steps (see Supplementary Figure 6 for details of steps) of the temperature-driven active learning-algorithm for MOF-5.

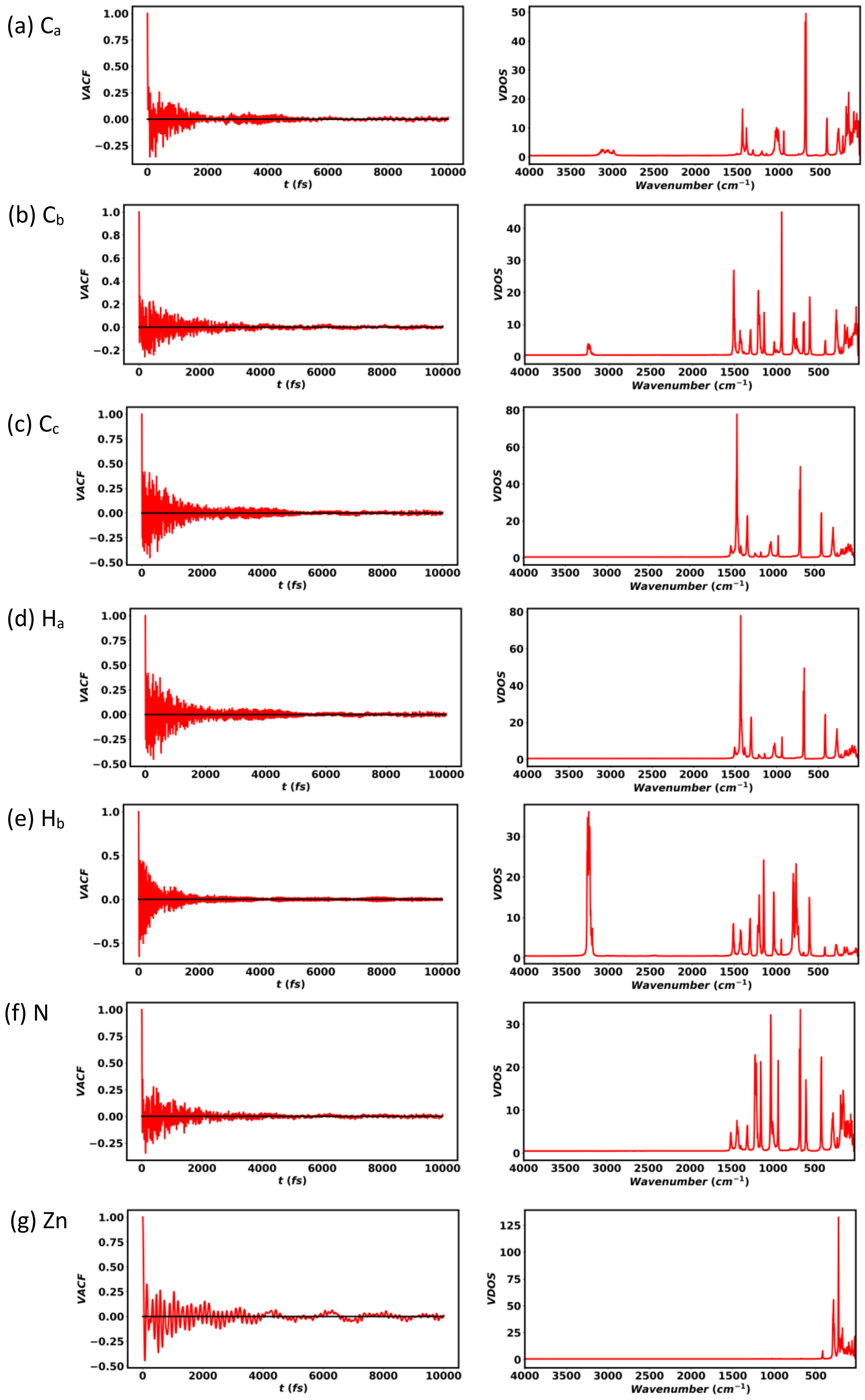


**Supplementary Figure 11:** Velocity autocorrelation function (left) and partial vibrational density of states (right) for each atom type of ZIF-8.

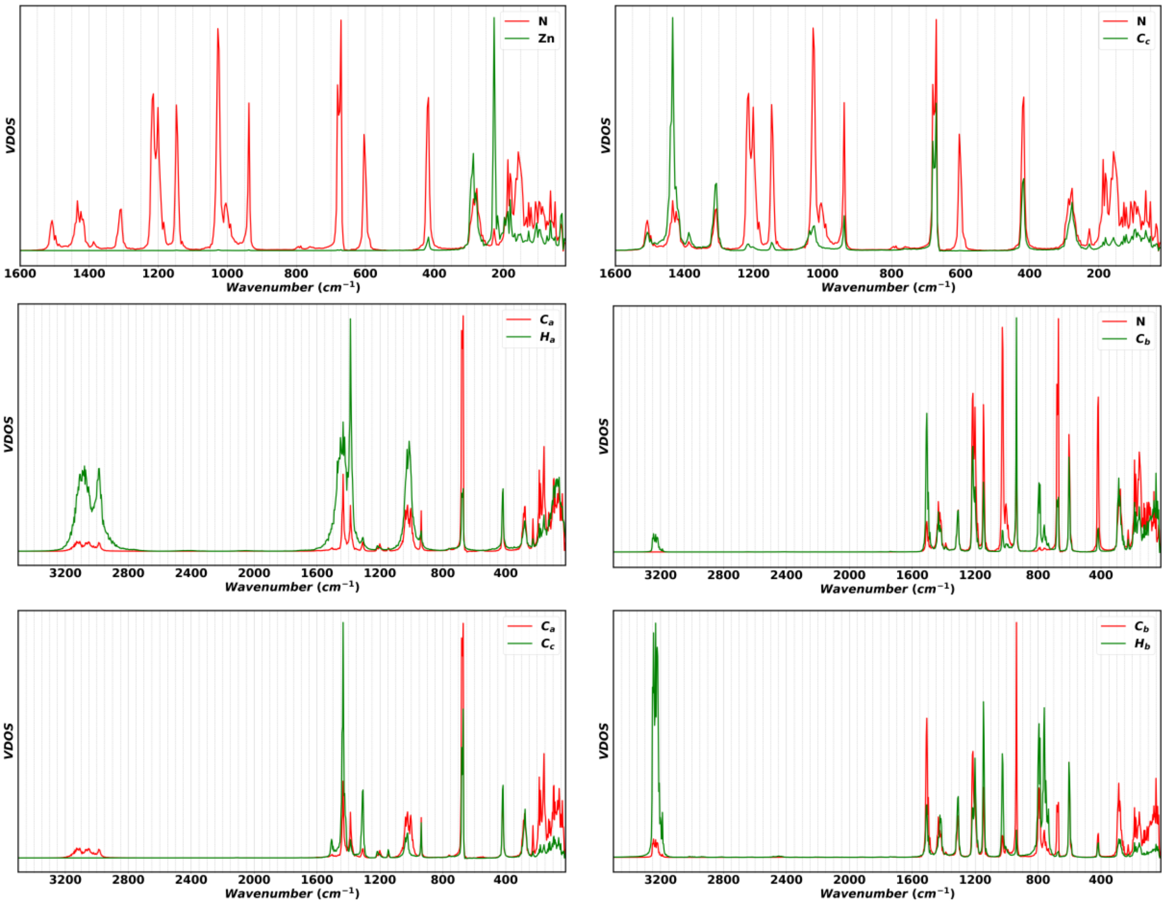


**Supplementary Figure 12:** Comparison of normalized partial vibrational density of states between neighbouring atoms of ZIF-8.

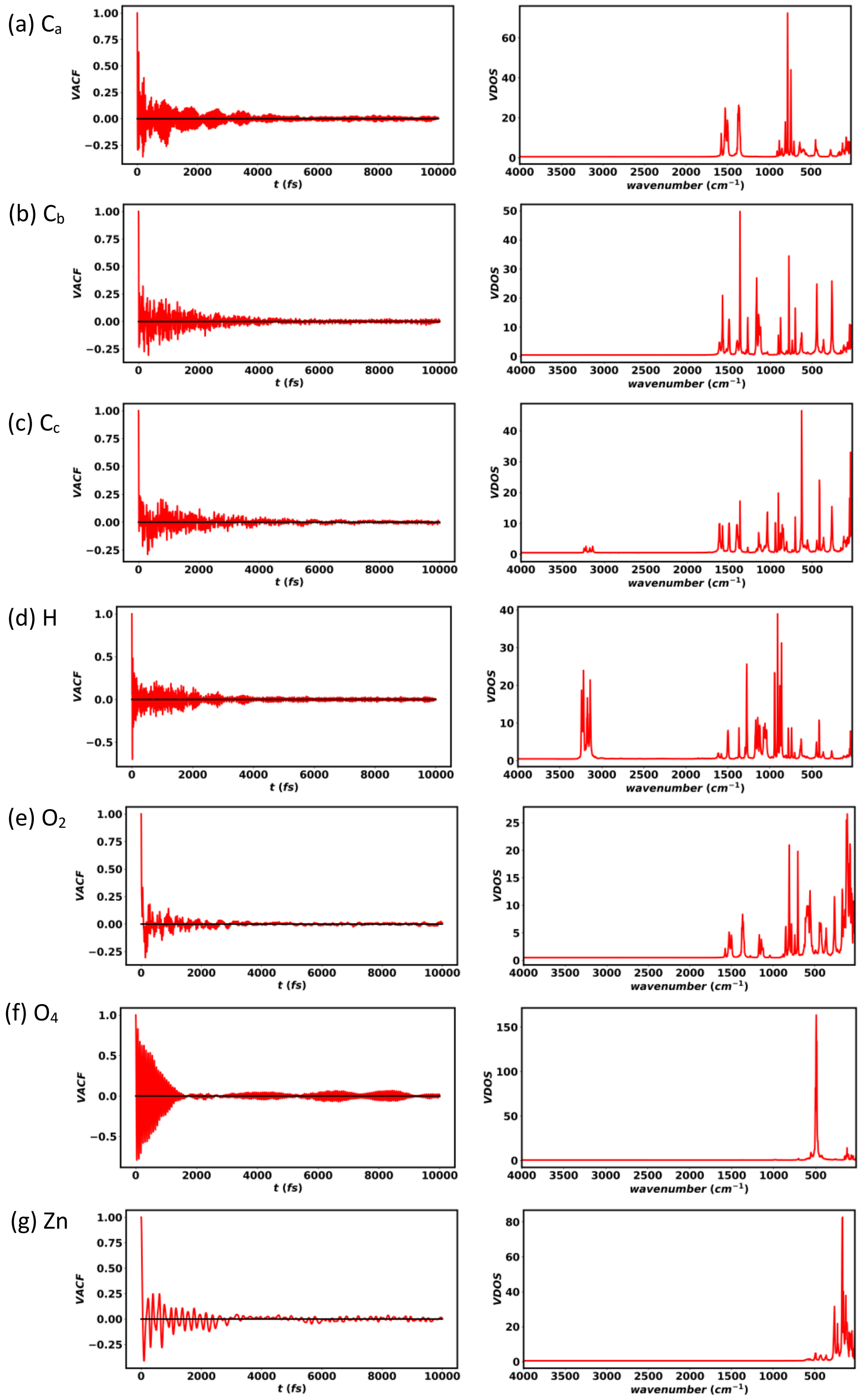


**Supplementary Figure 13:** Velocity autocorrelation function (left) and partial vibrational density of states (right) for each atom type of MOF-5.

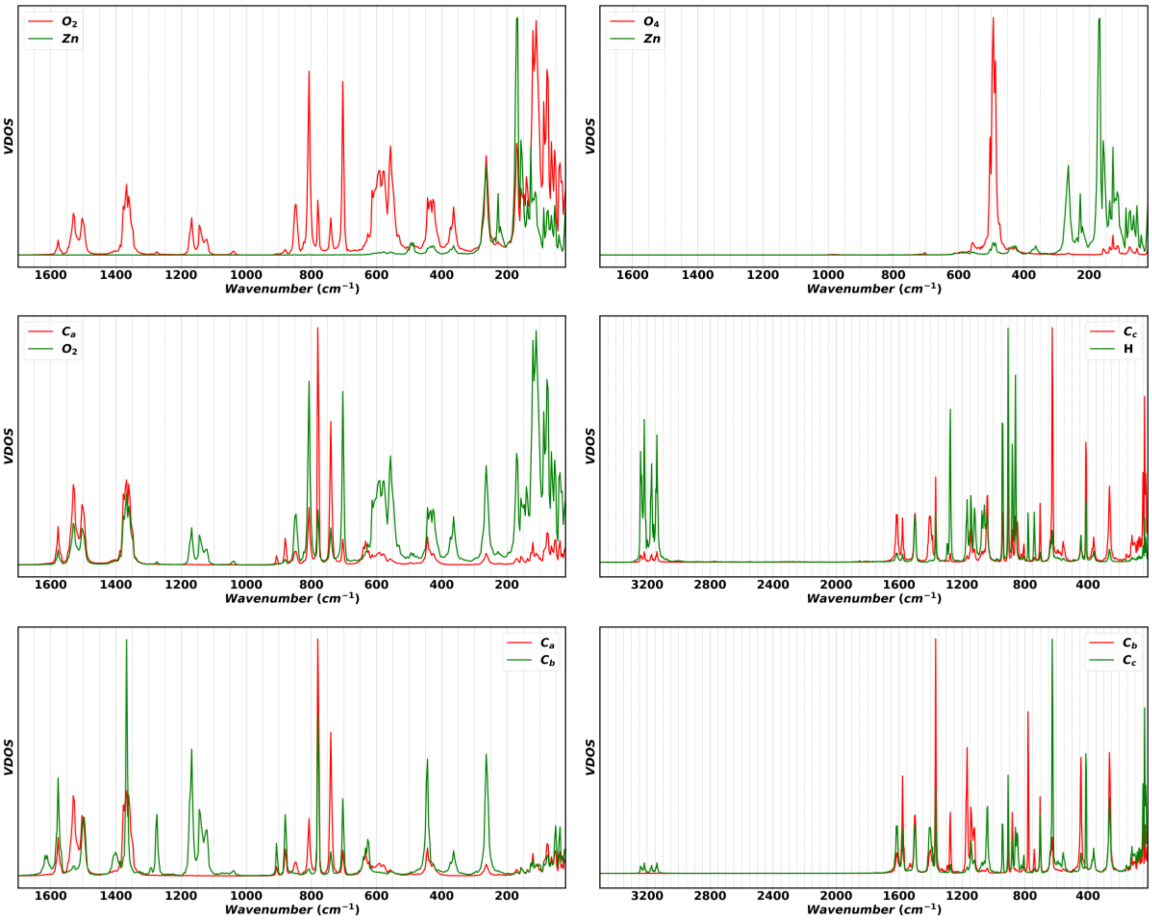


**Supplementary Figure 14:** Comparison of normalized partial vibrational density of states between neighbouring atoms of MOF-5.

## Supplementary Note 3: Well-tempered metadynamics simulation

Distribution of dihedral angles collective variables in a well-tempered metadynamics simulation at 250 K is shown in Supplementary Figure 15. Convergence of such simulations is identified by monitoring the free-energy barriers in different part of free-energy landscape as a function of simulation time. Plateau in (d) of Supplementary Figure 15 after 5 ns indicates the convergence of well-tempered metadynamics simulation.

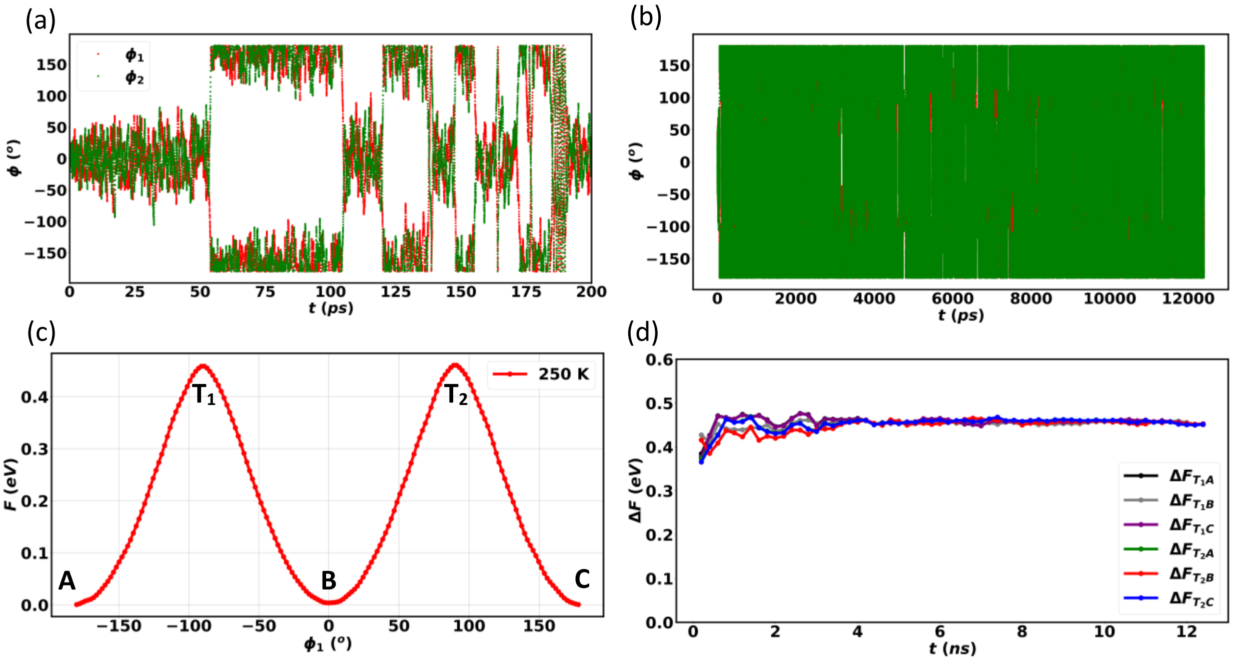


**Supplementary Figure 15:** Distribution of dihedral angle collective variables (in well-tempered metadynamics simulation at 250 K) as a function of simulation time of (a) 200 ps and (b) 12000 ps. (c) Free energy surface as a function of one collective variable. **A**, **B**, and **C** denotes stable states and $\mathbf{T_1}$ and $\mathbf{T_2}$ denotes transition states. (d) Evolution of rotational barrier heights (free energy difference between stable and transition states) as a function of simulation time.

## Supplementary Video 1

The file “Supplementary_Video_1.mp4” contains a movie which shows rotation of a phenylene ring in the MOF-5 during a well-tempered metadynamics simulation.

## Supplementary References


(1) MOF_MLP_2024 https://github.com/asharma-ms/MOF_MLP_2024.

(2) Agarwal, R. A.; Gupta, A. K.; De, D. Flexible Zn-MOF Exhibiting Selective CO2 Adsorption and Efficient Lewis Acidic Catalytic Activity. *Cryst. Growth Des.* **2019**, *19* (3), 2010–2018. https://doi.org/10.1021/acs.cgd.8b01462.

(3) Liang, W.; Bhatt, P. M.; Shkurenko, A.; Adil, K.; Mouchaham, G.; Aggarwal, H.; Mallick, A.; Jamal, A.; Belmabkhout, Y.; Eddaoudi, M. A Tailor-Made Interpenetrated MOF with Exceptional Carbon-Capture Performance from Flue Gas. *Chem* **2019**, *5* (4), 950–963. https://doi.org/https://doi.org/10.1016/j.chempr.2019.02.007.
